# 5G mobile networks and health—a state-of-the-science review of the research into low-level RF fields above 6 GHz

**DOI:** 10.1038/s41370-021-00297-6

**Published:** 2021-03-16

**Authors:** Ken Karipidis, Rohan Mate, David Urban, Rick Tinker, Andrew Wood

**Affiliations:** 1 Australian Radiation Protection and Nuclear Safety Agency, Melbourne, VIC Australia; 2 School of Health Sciences, Swinburne University of Technology, Melbourne, VIC Australia

**Keywords:** Radiation, Disease, Epidemiology, Health studies

## Abstract

The increased use of radiofrequency (RF) fields above 6 GHz, particularly for the 5 G mobile phone network, has given rise to public concern about any possible adverse effects to human health. Public exposure to RF fields from 5 G and other sources is below the human exposure limits specified by the International Commission on Non-Ionizing Radiation Protection (ICNIRP). This state-of-the science review examined the research into the biological and health effects of RF fields above 6 GHz at exposure levels below the ICNIRP occupational limits. The review included 107 experimental studies that investigated various bioeffects including genotoxicity, cell proliferation, gene expression, cell signalling, membrane function and other effects. Reported bioeffects were generally not independently replicated and the majority of the studies employed low quality methods of exposure assessment and control. Effects due to heating from high RF energy deposition cannot be excluded from many of the results. The review also included 31 epidemiological studies that investigated exposure to radar, which uses RF fields above 6 GHz similar to 5 G. The epidemiological studies showed little evidence of health effects including cancer at different sites, effects on reproduction and other diseases. This review showed no confirmed evidence that low-level RF fields above 6 GHz such as those used by the 5 G network are hazardous to human health. Future experimental studies should improve the experimental design with particular attention to dosimetry and temperature control. Future epidemiological studies should continue to monitor long-term health effects in the population related to wireless telecommunications.

## Introduction

There are continually emerging technologies that use radiofrequency (RF) electromagnetic fields particularly in telecommunications. Most telecommunication sources currently operate at frequencies below 6 GHz, including radio and TV broadcasting and wireless sources such as local area networks and mobile telephony. With the increasing demand for higher data rates, better quality of service and lower latency to users, future wireless telecommunication sources are planned to operate at frequencies above 6 GHz and into the ‘millimetre wave’ range (30–300 GHz) [1]. Frequencies above 6 GHz have been in use for many years in various applications such as radar, microwave links, airport security screening and in medicine for therapeutic applications. However, the planned use of millimetre waves by future wireless telecommunications, particularly the 5th generation (5 G) of mobile networks, has given rise to public concern about any possible adverse effects to human health.

The interaction mechanisms of RF fields with the human body have been extensively described and tissue heating is the main effect for RF fields above 100 kHz (e.g. HPA; SCENHIR) [2, 3]. RF fields become less penetrating into body tissue with increasing frequency and for frequencies above 6 GHz the depth of penetration is relatively short with surface heating being the predominant effect [4].

International exposure guidelines for RF fields have been developed on the basis of current scientific knowledge to ensure that RF exposure is not harmful to human health [5, 6]. The guidelines developed by the International Commission on Non-Ionizing Radiation Protection (ICNIRP) in particular form the basis for regulations in the majority of countries worldwide [7]. In the frequency range above 6 GHz and up to 300 GHz the ICNIRP guidelines prevent excessive heating at the surface of the skin and in the eye.

Although not as extensively studied as RF fields at lower frequencies, a number of studies have investigated the effects of RF fields at frequencies above 6 GHz. Previous reviews have reported studies investigating frequencies above 6 GHz that show effects although many of the reported effects occurred at levels greater than the ICNIRP guidelines [1, 8]. Given the public concern over the planned roll-out of 5 G using millimetre waves, it is important to determine whether there are any related adverse health consequences at levels encountered in the environment. The aim of this paper is to present a state-of-the-science review of the bioeffects research into RF fields above 6 GHz at low levels of exposure (exposure below the occupational limits of the ICNIRP guidelines). A meta-analysis of in vitro and in vivo studies, providing quantitative effect estimates for each study, is presented separately in a companion paper [9].

## Methods

The state-of-the-science review included a comprehensive search of all available literature and examined the extent, range and nature of evidence into the bioeffects of RF fields above 6 GHz, at levels below the ICNIRP occupational limits. The review consisted of biomedical studies on low-level RF electromagnetic fields from 6 GHz to 300 GHz published at any starting date up to December 2019. Studies were initially found by searching the databases PubMed, EMF-Portal, Google Scholar, Embase and Web of Science using the search terms “millimeter wave”, “millimetre wave”, “gigahertz”, “GHz” and “radar”. We further searched major reviews published by health authorities on RF and health [2, 3, 10, 11]. Finally, we searched the reference list of all the studies included. Studies were only included if the full paper was available in English.

Although over 300 studies were considered, this review was limited to experimental studies (in vitro, in vivo, human) where the stated RF exposure level was at or below the occupational whole-body limits specified by the ICNIRP (2020) guidelines: power density (PD) reference level of 50 W/m^2^ or specific absorption rate (SAR) basic restriction of 0.4 W/kg. Since the PD occupational limits for local exposure are more relevant to in vitro studies, and since these limits are higher, we have included those studies with PD up to 100–200 W/m^2^, depending on frequency. The review included studies below the ICNIRP general public limits that are lower than the occupational limits.

The review also included epidemiological studies (cohort, case-control, cross-sectional) investigating exposure to radar but excluded studies where the stated radar frequencies were below 6 GHz. Epidemiological studies on radar were included as they represent occupational exposure below the ICNIRP guidelines. Case reports or case series were excluded. Studies investigating therapeutical outcomes were also excluded unless they reported specific bio-effects.

The state-of-the-science review appraised the quality of the included studies, but unlike a systematic review it did not exclude any studies based on quality. The review also identified gaps in knowledge for future investigation and research. The reporting of results in this paper is narrative with tabular accompaniment showing study characteristics. In this paper, the acronym “MMWs” (or millimetre waves) is used to denote RF fields above 6 GHz.

## Results

The review included 107 experimental studies (91 in vitro, 15 in vivo, and 1 human) that investigated various bioeffects, including genotoxicity, cell proliferation, gene expression, cell signalling, membrane function and other effects. The exposure characteristics and biological system investigated in experimental studies for the various bioeffects are shown in Tables 1–6. The results of the meta-analysis of the in vitro and in vivo studies are presented separately in Wood et al. [9].

**Table 1:** Experimental studies investigating low-level RF fields above 6 GHz and genotoxicity.

Reference	Biological system	Frequency range	Intensity	Exposure duration	Results	Quality
[26] Crouzier et al.	Bacteria & Yeast	9 GHz	0.5 to 16 W/kg	20 min	No change in ROS production at low exposure levels. SAR above the limit	No blinding
[18] De Amicis et al.	Cells in culture	100–150 GHz	4 W/m^2^	Up to 24 h	No DNA damage but an increased occurrence of micro-nucleation. SAR above limit	Inadequate dosimetry and no blinding
[19] Franchini et al.	Cells in culture	25 GHz	8 W/m^2^	Up to 24 h	No DNA damage but an increased occurrence of micro-nucleation. SAR above limit	No blinding
[32] Gapeyev et al.	Cells in culture	42 GHz	1 W/m²	20 min	MMW pre-exposure reduced DNA damage after x-ray exposure to leucocytes	Poor temperature control
[33] Gapeyev and Lukyanova	Cells in culture	42 GHz	1 W/m²	20 min	MMW pre-exposure reduced DNA damage after x-ray exposure to leucocytes	Poor temperature control
[12] Garaj-Vrhovac et al.	Cells in culture	7 GHz	5–300 W/m²	10–60 min	No statistically significant increase in chromosome aberrations	Inadequate dosimetry and no blinding
[13] Garaj-Vrhovac et al.	Cells in culture	7 GHz	5–300 W/m²	10–60 min	No statistically significant increase in chromosome aberrations	Inadequate dosimetry and no blinding
[30] Hintzsche et al.	Cells in culture	106 GHz	0.43–43 W/m²	5 h	Increase in spindle disturbances, but no indication of structural chromosome aberrations	Well designed
[15] Hintzsche et al.	Cells in culture	106 GHz	0.4–20 W/m²	2–24 h	No DNA strand breaks or chromosome damage. SAR above limit	Inadequate temperature and sham control
[29] Kalantaryan et al.	Miscellaneous	65 GHz	0.5 W/m²	Up to 120 min	Changes in DNA strand separation during artificial synthesis	Poor dosimetry and temperature control
[24] Kesari and Behari	In vivo	50 GHz	0.0086 W/m^2^	2 h/day for 45 days	Increase in DNA double-strand breaks and a decrease in the levels of Protein kinase C	Low animal numbers (6 exposed)
[14] Korenstein-Ilan et al.	Cells in culture	100 GHz	0.31 W/m²	1–24 h	Chromosomal changes and asynchronous centromeres replications. SAR above limit	No blinding
[16] Koyama et al.	Cells in culture	60 GHz	10 W/m²	24 h	No increase in DNA strand breaks or heat shock protein expression	Well designed
[17] Koyama et al.	Cells in culture	45 GHz	10 W/m²	24 h	No increase in mironucleation, DNA strand breaks or heat shock protein expression	No blinding
[25] Kumar et al.	In vivo	10 and 50 GHz	2.1 W/m^2^	2 h/day for 45 days	Increase in ROS and increases and decreases in enzymes that control the build-up of ROS	Low animal numbers (6 exposed) and no blinding
[28] Lukashevsky and Belyaev	Bacteria & Yeast	69–71 GHz	Up to 5 W/m²	30 min	Increase in indicators of DNA damage. SAR above limit	Inadequate dosimetry and temperature control
[23] Paulraj and Behari	In vivo	16.5 GHz	10 W/m^2^	2 h/day for 35 days	Increase in indicators of DNA damage. SAR above limit	Low animal numbers (6 exposed) and no blinding
[20] Shckorbatov et al.	Cells in culture	42 GHz	2 W/m²	1–60 s	Decreased nuclei electrical charge and increased chromatin condensation in the nuclei	No blinding, sham control not described
[21] Shckorbatov et al.	Cells in culture	35 GHz	0.3 W/m^2^	10 s	Increase in chromatin condensation as indicated by an increase in heterochromatin granule quantity	Inadequate dosimetry and temperature control
[22] Shckorbatov et al.	Cells in culture	36 GHz	0.01–1 W/m^2^	1–10 s	Increase in chromatin condensation as indicated by an increase in heterochromatin granule quantity. SAR above limit	Inadequate dosimetry and temperature control
[27] Smolyanskaya and Vilenskaya	Bacteria & Yeast	45–46 GHz	0.1–10 W/m^2^	0.5–2 h	Increase in indicator of DNA damage	Statistical methods and dosimetry were not described
[31] Zeni et al.	Cells in culture	120–130 GHz	0.5–2.3 W/m²	20 min	No indication of DNA damage or changes in cell cycle kinetics. SAR above limit	Inadequate temperature control

**Table 2:** Experimental studies investigating low-level RF fields above 6 GHz and cell proliferation.

Reference	Biological system	Frequency range	Intensity	Exposure duration	Results	Quality
[56] Badzhinyan et al.	Cells in culture	40–90 GHz	0.5–1000 W/m^2^	8 min	No change in cell survival at exposure levels below the limits	Inadequate dosimetry and temperature control
[51] Beneduci et al.	Cells in culture	53–78 GHz	1 µW, 44–46 mW	1–3 h/day for 5–10 days	Reduced cancer cell proliferation and changes in cell morphology	Inadequate dosimetry and temperature control
[53] Beneduci et al.	Cells in culture	53–78 GHz	0.0007 W/m^2^	1–3 h/day for 5–10 days	Reduced cancer cell proliferation and changes in cell morphology	Inadequate dosimetry and temperature control
[54] Beneduci et al.	Cells in culture	53–78 GHz	0.01 W/m^2^	1 h/day for 4 days	Reduction in viable cancer cells and changes in cell structural morphology	Inadequate dosimetry and temperature control
[53] Beneduci	Cells in culture	42–54 GHz	1.1–3.7 W/m^2^	1 h/day for 4 days	No evidence of anti-proliferation effects in exposed cancer cells	Inadequate dosimetry and poor temperature control
[50] Chidichimo et al.	Cells in culture	53–78 GHz	7×10^–4^ W/m^2^	1 h/day for 12 days	Unclear results due to the in text results not matching supporting conclusions	Poor temperature control and no blinding
[38] Cohen et al.	Bacteria & Yeast	99 GHz	2 W/m^2^	1–19 h	No statistically significant changes in cell proliferation or survival. SAR above limit	No blinding
[48] Furia et al.	Bacteria & Yeast	42 GHz	Up to 0.08 W	Up to 4 h	No change in cell proliferation or viability	No blinding
[49] Gos et al.	Bacteria & yeast	40–43 GHz	0.005–0.5 W/m^2^	2 and 5.5 h	No changes in cell proliferation	Inadequate sham control and no blinding
[47] Grundler and Keilmann	Bacteria & Yeast	42 GHz	40 mW	NS	Enhanced and inhibited rates of cell proliferation	Inadequate dosimetry, statistical analysis not described
[46] Grundler and Keilmann	Bacteria & Yeast	42 GHz	1–20 W/m²	Up to 12 h	Enhanced and inhibited rates of cell proliferation	Inadequate sham control and no blinding
[45] Hovnanyan et al.	Bacteria & Yeast	51–53 GHz	0.6 W/m^2^	Up to 2 h	Increase in cell diameter and inhibition of cell growth	Inadequate dosimetry and temperature control
[37] Pakhomova et al.	Bacteria & Yeast	61–62 GHz	1.3 W/m^2^	30 min	MMW pre-exposure did not change cell survival or alter the frequency of mutations. SAR above limit	Inadequate temperature control
[36] Rojavin and Ziskin	Bacteria & Yeast	61 GHz	10 W/m²	Up to 1 h	Increase in cell survival if MMW exposure occurred after UVC exposure. No effect of MMW exposure alone. SAR above limit	No blinding
[57] Shiina et al.	Neural activity	60 GHz	10 W/m²	24 h	No change in neurite outgrowth	No blinding
[44] Soghomonyan and Trchounian	Bacteria & Yeast	51–53 GHz	0.6 W/m²	1 h	Changes in ion transport across the membrane and an inhibitory effect on bacteria proliferation and survival	Inadequate dosimetry and no blinding
[39] Tadevosyan et al.	Bacteria & Yeast	51–53 GHz	0.6 W/m^2^	Up to 1 h	Changes in ion transport across the membrane and an inhibitory effect on bacteria proliferation	Inadequate dosimetry and temperature control
[40] Torgomyan and Trchounian	Bacteria & Yeast	70–73 GHz	0.6 W/m^2^	Up to 1 h	Inhibition of proliferation and changes in membrane proteins	Inadequate dosimetry and temperature control
[41] Torgomyan et al.	Bacteria & Yeast	70–73 GHz	0.6 W/m^2^	Up to 2 h	Effect on bacterial growth and surrounding water medium	Inadequate dosimetry and temperature control
[42] Torgomyan et al.	Bacteria & Yeast	51–73 GHz	0.6 W/m^2^	1 h	Enhanced inhibitory effect of antibiotics on bacterial proliferation. Changes in ion transport	Inadequate dosimetry and temperature control
[43] Torgomyan et al.	Bacteria & Yeast	51–53 GHz	0.6 W/m^2^	1 h	Changes in the bacterial proliferation and survival. Changes in ion transport	Inadequate dosimetry and temperature control
[34] Webb and Booth	Bacteria & Yeast	65–75 GHz	NS	NS	Inhibition and stimulation of bacterial growth at specific frequencies	No details on dosimetry and no blinding
[35] Webb and Dodds	Bacteria & Yeast	136 GHz	7×10^–6^ W	Up to 4 h	Inhibition and stimulation of bacterial growth at specific frequencies	No details on dosimetry and no blinding
[55] Yaekashiwa et al.	Cells in culture	70–300 GHz	Up to 0.0127 W/m²	3–94 h	No change in proliferation, cell activity or cytotoxicity	No blinding

*NS* Not stated in the study.

**Table 3:** Experimental studies investigating low-level RF fields above 6 GHz and gene expression.

Reference	Biological system	Frequency range	Intensity	Exposure duration	Results	Quality
[64] Belyaev et al.	Bacteria & Yeast	41–52 GHz	0.01–1 W/m²	5–10 min	Frequency dependant changes in DNA conformation based on AVTD method and changes in DNA repair	Inadequate dosimetry and temperature control
[65] Belyaev et al.	Bacteria & Yeast	52 GHz	1 W/m²	5–10 min	Frequency dependant changes in DNA conformation based on AVTD method and changes in DNA repair	Inadequate dosimetry and temperature control
[66] Belyaev et al.	Bacteria & Yeast	41–52 GHz	0.01–3 W/m^2^	30 min	Frequency dependant changes in DNA conformation based on AVTD method and changes in DNA repair	Inadequate dosimetry and temperature control
[67] Belyaev et al.	Bacteria & Yeast	41–52 GHz	0.1–1 W/m^2^	5–10 min	Frequency dependant changes in DNA conformation based on AVTD method and changes in DNA repair	Inadequate dosimetry and temperature control
[68] Belyaev et al.	Bacteria & Yeast	41–52 GHz	10^16^–10^−6^ W/m^2^	10 min	Frequency dependant changes in DNA conformation based on AVTD method and changes in DNA repair	Inadequate dosimetry and temperature control
[69] Belyaev et al.	Bacteria & Yeast	41–52 GHz	0.1–1 W/m^2^	5 min	Frequency dependant changes in DNA conformation based on AVTD method and suppression of DNA repair	Inadequate dosimetry and temperature control
[71] Belyaev and Kravchenko	Cells in culture	41 GHz	10^−7^ – 1 W/m^2^	10 min	Frequency dependant changes in DNA conformation based on AVTD method. SAR above limit	Inadequate dosimetry and temperature control
[72] Belyaev et al.	Bacteria & Yeast	41–52 GHz	10^-16^ – 1 W/m^2^	10–50 min	Frequency dependant changes in DNA conformation based on AVTD method and changes in cell developmental dynamics	Inadequate dosimetry and temperature control
[72] Belyaev et al.	Bacteria & Yeast	52 GHz	10^−19^ – 0.003 W/m^2^	10 min	Frequency dependant changes in DNA conformation based on AVTD method	Inadequate dosimetry and temperature control
[76] Bush et al.	Cells in culture	38–75 GHz	Up to 5840 W/m^2^	15 min	No changes in protein synthesis and no resonance effects detected even at high exposure levels	Temperature control and dosimetry methods were not described
[75] Gandhi et al.	Bacteria & Yeast	26.5–90.0 GHz	Up to 3000 W/m^2^	Up to 5 s	No resonance effects detected even at exposure levels above the limits	Statistical methods not described
[58] Le Quement et al.	Cells in culture	60 GHz	18 W/m^2^	1–24 h	Five genes were reported to have transient expression changes after exposure. SAR above limit	No blinding, poor temperature control
[62] Nicolaz et al.	Cells in culture	60 GHz	1.4 W/m²	24–72 h	No change in ER homeostasis, protein folding, secretions or transcription factors	No blinding
[63] Nicolaz et al.	Cells in culture	59–61 GHz	0.9–1.4 W/m²	24 h.	No changes in mRNA expression of chaperone proteins. SAR above limit	No blinding
[73] Shcheglov et al.	Bacteria & Yeast	51 GHz	Up to 10^–7^ W/m^2^	10 min	Frequency dependant changes in DNA conformation. Cell to cell communication reported to enhance this effect	Inadequate dosimetry and temperature control
[74] Shcheglov et al.	Bacteria & Yeast	52 GHz	10^−14^ – 10 W/m²	Up to 10 min	Frequency dependant changes in DNA conformation. Cell to cell communication reported to enhance this effect	Inadequate dosimetry and temperature control
[59] Zhadobov et al.	Cells in culture	60 GHz	2.7 W/m²	1–33 h	No change in the expression of stress sensitive genes	Inadequate temperature control and no blinding
[60] Zhadobov et al.	Cells in culture	60 GHz	0.054–5.4 W/m²	1–33 h	No change in expression of chaperone proteins, heat shock proteins or reporting genes	No blinding
[61] Zhadobov et al.	Cells in culture	60 GHz	10 W/m²	24 h	No change in protein conformation, gene expression, cell viability or cell growth. SAR above limit	Temperature control not described and no blinding

**Table 4:** Experimental studies investigating low-level RF fields above 6 GHz and cell signalling and electrical activity.

Reference	Biological system	Frequency range	Intensity	Exposure duration	Results	Quality
[79] Minasyan et al.	Neural activity	38–54 GHz	4.8 W/m²	20–60 min	Change in the duration of the inter-spike intervals	Inadequate dosimetry and temperature control
[81] Munemori and Ikeda	Neural activity	10 GHz	2.5 W/m²	4 min	Increase and decrease in the variance of inter-spike intervals.	No sham control and poor temperature control
[82] Munemori and Ikeda	Neural activity	10 GHz	0.007–700 W/m²	1 min	Decrease in the distribution of the inter-spike intervals with increasing exposure levels	No sham control and poor temperature control
[83] Pakhomov et al.	Neural activity	40–52 GHz	2.4–30 W/m²	10 or 60 min	Reduction in the latency period and an increase in amplitude of CAPs	No blinding
[84] Pakhomov et al.,	Neural activity	40 GHz	0.2–26 W/m²	23 min	Reduction in the effect of high rate stimulus causing a decrease in the test CAP	No blinding
[85] Pakhomov et al.	Neural activity	40–50 GHz	2.5–25 W/m²	12–50 min	Reduction in the effect of high rate stimulus causing a decrease in the test CAP	No blinding
[86] Pikov and Siegel	Neural activity	60 GHz	0.00071–6 W/m²	NS	Reduced neuron firing rate and a decrease in input resistance	No blinding
[80] Pikov et al.	Neural activity	60 GHz	Up to 0.008 W/m²	1 min	Reduced neuron firing rate and a decrease in input resistance	No blinding
[87] Romanenko et al.	Neural activity	17–60 GHz	9–140 W/m²	60 s	Reduction in the action potential firing rate	No blinding
[88] Romanenko et al.	Neural activity	60 GHz	10–40 W/m²	60 s	Reduction in the action potential firing rate	No blinding

*NS* Not stated in the study.

**Table 5:** Experimental studies investigating low-level RF fields above 6 GHz and membrane effects.

Reference	Biological system	Frequency range	Intensity	Exposure duration	Results	Quality
[89] Beneduci et al.	Artificial cell suspensions	53–78 GHz	Up to 0.0027 W/kg	4 h	Delays in the transition from gel to liquid phase or vice versa	Statistical methods were not described and no blinding
[90] Beneduci et al.	Artificial cell suspensions	53–78 GHz	Up to 0.1 W/ m^2^	4 h	Reduction in water quadrupole splitting on simulated membrane	Statistical methods were not described and no blinding
[91] Beneduci et al.	Artificial cell suspensions	53–78 GHz	< 0.03 W/m^2^	Up to 40 h	Delays in the transition from gel to liquid phase or vice versa	Statistical methods were not described and no blinding
[93] Chen et al.	Miscellaneous	30 GHz	10–35 W/m^2^	1 h	Exposure increased membrane permeability	No sham control
[100] Cosentino et al.	Artificial cell suspensions	52–72 GHz	Up to 0.1 W/m^2^	Up to 4 h	Change in size due to osmotic stress and a decrease in water permeability	Inadequate dosimetry and poor temperature control
[97] D’Agostino et al.	Artificial cell suspensions	53 GHz	1.1 W/kg	Up to 30 min	Enhanced efflux of potassium from vesicles with increased amplitude of the electrical signals	Statistical methods were not described and no blinding
[96] Deghoyan et al.	Miscellaneous	90–160 GHz	1.49 W/kg	Up to 10 min	Decrease in the cell volume of neurons and rat brain tissue	Inadequate dosimetry and temperature control
[99] Di Donato et al.	Artificial cell suspensions	53 GHz	Up to 1 W/m^2^	Up to 2 min	Enhancement of the CA reaction rate resulting in membrane permeability changes	No blinding
[92] Geletyuk et al.	Cells in culture	42 GHz	1 W/m^2^	Up to 30 min	Changes in binding affinity of channels for calcium with associated lowering of channel opening probability	No sham, dosimetry description or temperature control
[45] Hovnanyan et al.	Bacteria & Yeast	51–53 GHz	0.6 W/m^2^	Up to 2 h	Increase in cell diameter and inhibition of cell growth	Inadequate dosimetry and temperature control
[98] Ramundo-Orlando et al.	Artificial cell suspensions	53 GHz	1 W/m^2^	Up to 10 min	Cell morphology changes i.e. elongation and diffusion of dye across the membrane	Statistical methods were not described and no blinding
[94] Shckorbatov et al.	Cells in culture	37 GHz	2 W/m^2^	1–60 s	Increase in cell permeability and both increased and decrease cell electronegativity	No sham and temperature control
[21] Shckorbatov et al.	Cells in culture	35 GHz	0.3 W/m^2^	10 s	Reported an indication of cell membrane damage	Inadequate dosimetry and temperature control
[44] Soghomonyan and Trchounian	Bacteria & Yeast	51–53 GHz	0.6 W/m²	1 h	Changes in ion transport across the membrane and inhibitory effect on bacteria proliferation and survival	Inadequate dosimetry and no blinding
[38] Tadevosyan et al.	Bacteria & Yeast	51–53 GHz	0.6 W/m^2^	Up to 1 h	Changes in ion transport across the membrane and inhibitory effect on bacteria proliferation	Inadequate dosimetry and poor temperature control
[40] Torgomyan and Trchounian	Bacteria & Yeast	70–73 GHz	0.6 W/m^2^	Up to 1 h	Inhibition of proliferation and changes in membrane proteins	Inadequate dosimetry and temperature control
[41] Torgomyan et al.	Bacteria & Yeast	70–73 GHz	0.6 W/m^2^	Up to 2 h	Effect on bacterial growth and changes in ion transport	Inadequate dosimetry and temperature control
[42] Torgomyan et al.	Bacteria & Yeast	51–73 GHz	0.6 W/m^2^	1 h	Enhanced the inhibitory effect of antibiotics on bacterial proliferation. Changes in ion transport	Inadequate dosimetry and temperature control
[43] Torgomyan et al.	Bacteria & Yeast	51–53 GHz	0.6 W/m^2^	1 h	Changes in the bacterial proliferation and survival. Changes in ion transport	Inadequate dosimetry and temperature control
[95] Zhadobov et al.	Artificial cell suspensions	60 GHz	Up to 9 W/m²	Up to 5 h	Increases in lateral membrane pressure but no changes to the microdomain organisation	Statistical analysis not described and no blinding

**Table 6:** Experimental studies investigating low-level RF fields above 6 GHz and other effects.

Reference	Biological system	Frequency range	Intensity	Exposure duration	Results	Quality
[117] Bellossi et al.	In vivo	60 GHz	5.1 W/m^2^	30 min/day to death	Increased survival for the leukaemia inoculated mice	No temperature control and sham controls
[106] Gapeyev et al.	Cells in culture	41 to 42 GHz	0.24–0.5 W/m²	20 min	Frequency dependant change in ROS production	Inadequate dosimetry and temperature control methods not described
[107] Gapeyev et al.	Cells in culture	41 to 42 GHz	0.24–2.4 W/m²	20 min	Frequency dependant change in ROS production	Inadequate dosimetry and poor temperature control
[110] Gapeyev et al.	Cells in culture	42 GHz	1 W/m²	20 min	Changes in fatty acid concentrations in thymus cells and blood plasma	Poor temperature control and no blinding
[111] Gapeyev et al.	Cells in culture	40 GHz	1 W/m²	20 min	Changes in fatty acid concentrations of tumour bearing mice and restoration of fatty acid levels in the thymus	Poor temperature control and no blinding
[112] Gapeyev et al.	Cells in culture	40 GHz	1 W/m²	20 min	Accelerated recovery of fatty acid after X-ray exposure	Poor temperature control and no blinding
[109] Homenko et al.	Miscellaneous	100 GHz	0.31 W/m²	1, 2 and 24 h	Reduction in enzyme activity and decreased stability of antigen antibody complexes	No blinding
[104] Kesari and Behari	In vivo	10 and 50 GHz	0.0086 W/m^2^	2 h/day for 45 days	Increase and decrease in enzymes that control the build-up of ROS. Changes in cell cycle kinetics	Low animal numbers (6 exposed)
[119] Khizhnyak and Ziskin	Miscellaneous	53–78 GHz	0.1 – 10000 W/m^2^	Up to 40 min	Temperature oscillations in the liquid medium. SAR above limit	Inadequate dosimetry, no sham control and no blinding
[105] Kumar et al.	In vivo	10 GHz	2.1 W/m^2^	2 h/day for 45 days	Decrease in the activity of histone kinase and an increase in ROS and the rate of apoptosis. There was also changes in cell cycle kinetics	Low animal numbers (6 exposed), no blinding
[101] Manikowska et al.	In vivo	9.4 GHz	10–100 W/m²	1 h/day for 2 weeks	Increase in occurrence of translocations and unpaired chromosomes during meiosis in sperm cells of mice	Inadequate dosimetry and temperature control
[114] Muller et al.	Human volunteers	77 GHz	0.03 W/m²	15 min	No alterations of autonomic nerve activity or cardiovascular function	Inadequate dosimetry and temperature control
[118] Olchowik and Maj	In vivo	53 GHz	10–100 W/ m^2^	20 min/day for 30 days	No effects below limit, above the limit the effect of hydrocortisone on gamma-glutamyl transpeptidase was blocked	No description of dosimetry and poor temperature control
[113] Rotkovská et al.	In vivo	34 GHz	0.2 W/m²	17 h/day for 10 days	Increase in progenitors of granulocytes and macrophages in the bone marrow of exposed mice	Poor temperature control and statistical analysis not described
[108] Safronova et al.	Cells in culture	42 GHz	0.195 W/m²	20 min	Enhanced response of primed neutrophils to a chemotactic peptide	No blinding and poor temperature control
[120] Sarapultseva et al.	Miscellaneous	1–10 GHz	0.05–0.5 W/ m^2^	Up to 10 h	Exposure decreased the motility of the protozoa S. ambiguum and their non-exposed offspring	Inadequate dosimetry and no blinding
[102] Subbotina et al.	In vivo	NS	3 W/m²	3.5–32 h for 63 days	Increase in the occurrence of abnormal sperm and an increase in litter size of exposed male mice	No description of dosimetry or temperature control
[116] Stensaas et al.	Cells in culture	41–74 GHz	Up to 10000 W/m^2^	1 h	No effect on the ultracellular structure of the cells when temperature was controlled	Inadequate dosimetry, statistical analysis not described
[103] Volkova et al.	Cells in culture	42 GHz	0.3 W/m²	5–15 min	No change to sperm membrane integrity or nuclear chromatin status. Increase in percentage of mobile sperm	Inadequate dosimetry and temperature control
[115] Webb and Booth	Cells in culture	66–76 GHz	2 × 10^−5^ – 0.000103 W	NS	Frequency specific differences in the attenuation of MMW in healthy and tumour cells	Inadequate dosimetry, no sham or temperature control

*NS* Not stated in the study.

### Genotoxicity

Studies have examined the effects of exposing whole human or mouse blood samples or lymphocytes and leucocytes to low-level MMWs to determine possible genotoxicity. Some of the genotoxicity studies have looked at the possible effects of MMWs on chromosome aberrations [12–14]. At exposure levels below the ICNIRP limits, the results have been inconsistent, with either a statistically significant increase [14] or no significant increase [12, 13] in chromosome aberrations.

MMWs do not penetrate past the skin therefore epithelial and skin cells have been a common model of examination for possible genotoxic effects. DNA damage in a number of epithelial and skin cell types and at varied exposure parameters both below and above the ICNIRP limits have been examined using comet assays [15–19]. Despite the varied exposure models and methods used, no statistically significant evidence of DNA damage was identified in these studies. Evidence of genotoxic damage was further assessed in skin cells by the occurrence of micro-nucleation. De Amicis et al. [18] and Franchini et al. [19] reported a statistically significant increase in micro-nucleation, however, Hintzsche et al. [15] and Koyama et al. [16, 17] did not find an effect. Two of the studies also examined telomere length and found no statistically significant difference between exposed and unexposed cells [15, 19]. Last, a Ukrainian research group examined different skin cell types in three studies and reported an increase in chromosome condensation in the nucleus [20–22]; these results have not been independently verified. Overall, there was no confirmed evidence of MMWs causing genotoxic damage in epithelial and skin cells.

Three studies from an Indian research group have examined indicators of DNA damage and reactive oxygen species (ROS) production in rats exposed in vivo to MMWs. The studies reported DNA strand breaks based on evidence from comet assays [23, 24] and changes in enzymes that control the build-up of ROS [24]. Kumar et al. also reported an increase in ROS production [25]. All the studies from this research group had low animal numbers (six animals exposed) and their results have not been independently replicated. An in vitro study that investigated ROS production in yeast cultures reported an increase in free radicals exposed to high-level but not low-level MMWs [26].

Other studies have looked at the effect of low-level MMWs on DNA in a range of different ways. Two studies reported that MMWs induce colicin synthesis and prophage induction in bacterial cells, both of which are suggested as indicative of DNA damage [27, 28]. Another study suggested that DNA exposed to MMWs undergoes polymerase chain reaction synthesis differently than unexposed DNA [29], although no statistical analysis was presented. Hintzsche et al. reported statistically significant occurrence of spindle disturbance in hybrid cells exposed to MMWs [30]. Zeni et al. found no evidence of DNA damage or alteration of cell cycle kinetics in blood cells exposed to MMWs [31]. Last, two studies from a Russian research group examined the protective effects of MMWs where mouse blood leukocytes were pre-exposed to low-level MMWs and then to X-rays [32, 33]. The studies reported that there was statistically significant less DNA damage in the leucocytes that were pre-exposed to MMWs than those exposed to X-rays alone. Overall, these studies had no independent replication.

### Cell proliferation

A number of studies have examined the effects of low-level MMWs on cell proliferation and they have used a variety of cellular models and methods of investigation. Studies have exposed bacterial cells to low-level MMWs alone or in conjunction with other agents. Two early studies reported changes in the growth rate of E. coli cultures exposed to low-level MMWs; however, both of these studies were preliminary in nature without appropriate dosimetry or statistical analysis [34, 35]. Two studies exposed E. coli cultures and one study exposed yeast cell cultures to MMWs alone, and before and after UVC exposure [36–38]. All three studies reported that MMWs alone had no significant effect on bacterial cell proliferation or survival. Rojavin et al., however, did report that when E. coli bacteria were exposed to MMWs after UVC sterilisation treatment, there was an increase in their survival rate [36]. The authors suggested this could be due to the MMW activation of bacterial DNA repair mechanisms. Other studies by an Armenian research group reported a reduction in E. coli cell growth when exposed to MMWs [39–45]. These studies reported that when E.coli cultures were exposed to MMWs in the presence of antibiotics, there was a greater reduction in the bacterial growth rate and an increase in the time between bacterial cell division compared with antibiotics exposure alone. Two of these studies investigated if these effects could be due to a reduction in the activity of the E. coli ATPase when exposed to MMWs. The studies reported exposure to MMWs in combination with particular antibiotics changed the concentration of H^+^ and K^+^ ions in the E.coli cells, which the authors linked to changes in ATPase activity [43, 44]. Overall, the results from studies on cell proliferation of bacterial cells have been inconsistent with different research groups reporting conflicting results.

Studies have also examined how exposure to low-level MMWs could affect cell proliferation in yeast. Two early studies by a German research group reported changes in yeast cell growth [46, 47]. However, another two independent studies did not report any changes in the growth rate of exposed yeast [48, 49]. Furia et al. [48] noted that the Grundler and Keilmann studies [46, 47] had a number of methodical issues, which may have skewed their results, such as poor exposure control and analysis of results. Another study exposed yeast to MMWs before and after UVC exposure and reported that MMWs did not change the rates of cell survival [37].

Studies have also examined the possible effect of low-level MMWs on tumour cells with some studies reporting a possible anti-proliferative effect. Chidichimo et al. reported a reduction in the growth of a variety of tumour cells exposed to MMWs; however, the results of the study did not support this conclusion [50]. An Italian research group published a number of studies investigating proliferation effects on human melanoma cell lines with conflicting results. Two of the studies reported reduced growth rate [51, 52] and a third study showed no change in proliferation or in the cell cycle [53]. Beneduci et al. also reported changes in the morphology of MMW exposed cells; however, the authors did not present quantitative data for these reported changes [51, 52]. In another study by the same Italian group, Beneduci et al. reported that exposure to low-level MMWs had a greater than 40% reduction in the number of viable erythromyeloid leukaemia cells compared with controls; however, there was no significant change in the number of dead cells [54]. More recently, Yaekashiwa et al. reported no statistically significant effect in proliferation or cellular activity in glioblastoma cells exposed to low-level MMWs [55].

Other studies did not report statistically significant effects on proliferation in chicken embryo cell cultures, rat nerve cells or human skin fibroblasts exposed to low-level MMWs [55–57].

### Gene expression

Some studies have investigated whether low-level MMWs can influence gene expression. Le Queument et al. examined a multitude of genes using microarray analyses and reported transient expression changes in five of them. However, the authors concluded that these results were extremely minor, especially when compared with studies using microarrays to study known pollutants [58]. Studies by a French research group have examined the effect of MMWs on stress sensitive genes, stress sensitive gene promotors and chaperone proteins in human glial cell lines. In two studies, glial cells were exposed to low-level MMWs and there was no observed modification in the expression of stress sensitive gene promotors when compared with sham exposed cells [59–61]. Further, glial cells were examined for the expression of the chaperone protein clusterin (CLU) and heat shock protein HSP70. These proteins are activated in times of cellular stress to maintain protein functions and help with the repair process [60]. There was no observed modification in gene expression of the chaperone proteins. Other studies have examined the endoplasmic reticulum of glial cells exposed to MMWs [62, 63]. The endoplasmic reticulum is the site of synthesis and folding of secreted proteins and has been shown to be sensitive to environmental insults [62]. The authors reported that there was no elevation in mRNA expression levels of endoplasmic reticulum specific chaperone proteins. Studies of stress sensitive genes in glial cells have consistently shown no modification due to low-level MMW exposure [59–63].

Belyaev and co-authors have studied a possible resonance effect of low-level MMWs primarily on Escherichia Coli (E. coli) cells and cultures. The Belyaev research group reported that the resonance effect of MMWs can change the conformation state of chromosomal DNA complexes [64–74]; however, most of these experiments were not temperature controlled. This resonance effect was not supported by earlier experiments on a number of different cell types conducted by Gandhi et al. and Bush et al. [75, 76].

The results of Belyaev and co-workers have primarily been based on evidence from the anomalous viscosity time dependence (AVTD) method [77]. The research group argued that changes in the AVTD curve can indicate changes to the DNA conformation state and DNA-protein bonds. Belyaev and co-workers have reported in a number of studies that differences in the AVTD curve were dependent on several parameter including MMW characteristics (frequency, exposure level, and polarisation), cellular concentration and cell growth rate [69, 71–74]. In some of the Belyaev studies E. coli were pre-exposed to X-rays, which was reported to change the AVTD curve; however, if the cells were then exposed to MMWs there was no longer a change in the AVTD curve [64–67]. The authors suggested that exposure to MMWs increased the rate of recovery in bacterial cells previously exposed to ionising radiation. The Belyaev group also used rat thymocytes in another study and they concluded that the results closely paralleled those found in E. coli cells [67]. The studies on the DNA conformation state change relied heavily on the AVTD method that has only been used by the Balyaev group and has not been independently validated [78].

### Cell signalling and electrical activity

Studies examining effects of low-level MMWs on cell signalling have mainly involved MMW exposure to nervous system tissue of various animals. An in vivo study on rats recorded extracellular background electrical spike activity from neurons in the supraoptic nucleus of the hypothalamus after MMW exposure [79]. The study reported that there were changes in inter-spike interval and spike activity in the cells of exposed animals when compared with controls. There was also a mixture of significant shifts in neuron population proportions and spike frequency. The effect on the regularity of neuron spike activity was greater at higher frequencies. An in vitro study on rat cortical tissue slices reported that neuron firing rates decreased in half of the samples exposed to low-level MMWs [80]. The width of the signals was also decreased but all effects were short lived. The observed changes were not consistent between the two studies, but this could be a consequence of different brain regions being studied.

In vitro experiments by a Japanese research group conducted on crayfish exposed the dissected optical components and brain to MMWs [81, 82]. Munemori and Ikeda reported that there was no significant change in the inter-spike intervals or amplitude of spontaneous discharges [81]. However, there was a change in the distribution of inter-spike intervals where the initial standard deviation decreased and then restored in a short time to a rhythm comparable to the control. A follow-up study on the same tissues and a wide range of exposure levels (many above the ICNIRP limits) reported similar results with the distribution of spike intervals decreasing with increasing exposure level [82]. These results on action potentials in crayfish tissue have not been independently investigated.

Mixed results were reported in experiments conducted by a US research group on sciatic frog nerve preparations. These studies applied electrical stimulation to the nerve and examined the effect of MMWs on the compound action potentials (CAPs) conductivity through the neurological tissue fibre. Pakhomov et al. found a reduction in CAP latency accompanied by an amplitude increase for MMWs above the ICNIRP limits but not for low-level MMWs [83]. However, in two follow-up studies, Pakhomov et al. reported that the attenuation in amplitude of test CAPs caused by high-rate stimulus was significantly reduced to the same magnitude at various MMW exposure levels [84, 85]. In all of these studies, the observed effect on the CAPs was temporal and reversible, but there were implications of a frequency specific resonance interaction with the nervous tissue. These results on action potentials in frog sciatic nerves have not been investigated by others.

Other common experimental systems involved low-level MMW exposure to isolated ganglia of leeches. Pikov and Siegel reported that there was a decrease in the firing rate in one of the tested neurons and, through the measurement of input resistance in an inserted electrode, there was a transient dose-dependent change in membrane permeability [86]. However, Romanenko et al. found that low-level MMWs did not cause suppression of neuron firing rate [87]. Further experiments by Romanenko et al. reported that MMWs at the ICNIRP public exposure limit and above reported similar action potential firing rate suppression [88]. Significant differences were reported between MMW effects and effects due to an equivalent rise in temperature caused by heating the bathing solution by conventional means.

### Membrane effects

Studies examining membrane interactions with low-level MMWs have all been conducted at frequencies above 40 GHz in in vitro experiments. A number of studies investigated membrane phase transitions involving exposure to a range of phospholipid vesicles prepared to mimic biological cell membranes. One group of studies by an Italian research group reported effects on membrane hydration dynamics and phase transition [89–91]. Observations included transition delays from the gel to liquid phase or vice versa when compared with sham exposures maintained at the same temperature; the effect was reversed after exposure. These reported changes remain unconfirmed by independent groups.

A number of studies investigated membrane permeability. One study focussed on Ca^2+^ activated K^+^ channels on the membrane surface of cultured kidney cells of African Green Marmosets [92]. The study reported modifications to the Hill coefficient and apparent affinity of the Ca^2+^ by the K^+^ channels. Another study reported that the effectiveness of a chemical to supress membrane permeability in the gap junction was transiently reduced when the cells were exposed to MMWs [93, 94]. Two studies by one research group reported increases in the movement of molecules into skin cells during MMW exposure and suggested this indicates increased cell membrane permeability [21, 91]. Permeability changes based on membrane pressure differences were also investigated in relation to phospholipid organisation [95]. Although there was no evidence of effects on phospholipid organisation on exposed model membranes, the authors reported a measurable difference in membrane pressure at low exposure levels. Another study reported neuron shrinkage and dehydration of brain tissues [96]. The study reported this was due to influences of low-level MMWs on the cellular bathing medium and intracellular water. Further, the authors suggested this influence of MMWs may have led to formation of unknown messengers, which are able to modulate brain cell hydration. A study using an artificial axon system consisting of a network of cells containing aqueous phospholipid vesicles reported permeability changes with exposure to MMWs by measuring K^+^ efflux [97]. In this case, the authors emphasised limitations in applying this model to processes within a living organism. The varied effects of low-level MMWs on membrane permeability lack replication.

Other studies have examined the shape or size of vesicles to determine possible effects on membrane permeability. Ramundo-Orlando et al., reported effects on the shape of giant unilamellar vesicles (GUVs), specifically elongation, attributed to permeability changes [98]. However, another study reported that only smaller diameter vesicles demonstrated a statistically significant change when exposed to MMWs [99]. A study by Cosentino et al. examined the effect of MMWs on the size distributions of both large unilamellar vesicles (LUVs) and GUVs in in vitro preparations [100]. It was reported that size distribution was only affected when the vesicles were under osmotic stress, resulting in a statistically significant reduction in their size. In this case, the effect was attributed to dehydration as a result of membrane permeability changes. There is, generally, lack of replication on physical changes to phospholipid vesicles due to low-level MMWs.

Studies on E. coli and E. hirae cultures have reported resonance effects on membrane proteins and phospholipid constituents or within the media suspension [39–42]. These studies observed cell proliferation effects such as changes to cell growth rate, viability and lag phase duration. These effects were reported to be more pronounced at specific MMW frequencies. The authors suggested this could be due to a resonance effect on the cell membrane or the suspension medium. Torgomyan et al. and Hovnanyan et al. reported similar changes to proliferation that they attributed to changes in membrane permeability from MMW exposure [43, 45]. These experiments were all conducted by an Armenian research group and have not been replicated by others.

### Other effects

A number of studies have reported on the experimental results of other effects. Reproductive effects were examined in three studies on mice, rats and human spermatozoa. An in vivo study on mice exposed to low-level MMWs reported that spermatogonial cells had significantly more metaphase translocation disturbances than controls and an increased number of cells with unpaired chromosomes [101]. Another in vivo study on rats reported increased morphological abnormalities to spermatozoa following exposure, however, there was no statistical analysis presented [102]. Conversely, an in vitro study on human spermatozoa reported that there was an increase in motility after a short time of exposure to MMWs with no changes in membrane integrity and no generation of apoptosis [103]. All three of these studies looked at different effects on spermatozoa making it difficult to make an overall conclusion. A further two studies exposed rats to MMWs and examined their sperm for indicators of ROS production. One study reported both increases and decreases in enzymes that control the build-up of ROS [104]. The other study reported a decrease in the activity of histone kinase and an increase in ROS [105]. Both studies had low animal numbers (six animals exposed) and these results have not been independently replicated.

Immune function was also examined in a limited number of studies focussing on the effects of low-level MMWs on antigens and antibody systems. Three studies by a Russian research group that exposed neutrophils to MMWs reported frequency dependant changes in ROS production [106–108]. Another study reported a statistically significant decrease in antigen binding to antibodies when exposed to MMWs [109]; the study also reported that exposure decreased the stability of previously formed antigen–antibody complexes.

The effect on fatty acid composition in mice exposed to MMWs has been examined by a Russian research group using a number of experimental methods [110–112]. One study that exposed mice afflicted with an inflammatory condition to low-level MMWs reported no change in the fatty acid concentrations in the blood plasma. However, there was a significant increase in the omega-3 and omega-6 polyunsaturated fatty acid content of the thymus [110]. Another study exposed tumour-bearing mice and reported that monounsaturated fatty acids decreased and polyunsaturated fatty acids increased in both the thymus and tumour tissue. These changes resulted in fatty acid composition of the thymus tissue more closely resembling that of the healthy control animals [111]. The authors also examined the effect of exposure to X-rays of healthy mice, which was reported to reduce the total weight of the thymus. However, when the thymus was exposed to MMWs before or after exposure to X-rays, the fatty acid content was restored and was no longer significantly different from controls [112]. Overall, the authors reported a potential protective effect of MMWs on the recovery of fatty acids, however, all the results came from the same research group with a lack of replication from others.

Physiological effects were examined by a study conducted on mice exposed to WWMs to assess the safety of police radar [113]. The authors reported no statistically significant changes in the physiological parameters tested, which included body mass and temperature, peripheral blood and the mass and cellular composition, and number of cells in several important organs. Another study exposing human volunteers to low-level MMWs specifically examined cardiovascular function of exposed and sham exposed groups by electrocardiogram (ECG) and atrioventricular conduction velocity derivation [114]. This study reported that there were no significant differences in the physiological indicators assessed in test subjects.

Other individual studies have looked at various other effects. An early study reported differences in the attenuation of MMWs at specific frequencies in healthy and tumour cells [115]. Another early study reported no effect in the morphology of BHK-21/C13 cell cultures when exposed to low-level MMWs; the study did report morphological changes at higher levels, which were related to heating [116]. One study examined whether low-level MMWs induced cancer promotion in leukaemia and Lewis tumour cell grafted mice. The study reported no statistically significant growth promotion in either of the grafted cancer cell types [117]. Another study looked at the activity of gamma-glutamyl transpeptidase enzyme in rats after treatment with hydrocortisone and exposure to MMWs [118]. The study reported no effects at exposures below the ICNIRP limit, however, at levels above authors reported a range of effects. Another study exposed saline liquid solutions to continuous low and high level MMWs and reported temperature oscillations within the liquid medium but lacked a statistical analysis [119]. Another study reported that low-level MMWs decrease the mobility of the protozoa S. ambiguum offspring [120]. None of the reported effects in all of these other studies have been investigated elsewhere.

### Epidemiological studies

There are no epidemiological studies that have directly investigated 5 G and potential health effects. There are however epidemiological studies that have looked at occupational exposure to radar, which could potentially include the frequency range from 6 to 300 GHz. Epidemiological studies on radar were included as they represent occupational exposure below the ICNIRP guidelines. The review included 31 epidemiological studies (8 cohort, 13 case-control, 9 cross-sectional and 1 meta-analysis) that investigated exposure to radar and various health outcomes including cancer at different sites, effects on reproduction and other diseases. The risk estimates as well as limitations of the epidemiological studies are shown in Table 7.

**Table 7:** Epidemiological studies investigating occupational exposure to radar at frequencies above 6 GHz.

Reference	Type of study	Study population	Exposure assessment	Disease	Risk Estimate	Limitations
[143] Baste et al.	Cross-sectional	Norwegian Navy personnel (10,497 men)	Self-reported	Infertility	OR 1.86 (1.46–2.37)	No information on confounding factors including sailing time
[147] Baste et al.	Cohort (retrospective)	Norwegian Navy personnel followed from 1967 to 2008 (28,337 men)	Job-exposure matrix	Perinatal mortality	Acute exposure OR 2.87 (1.25–6.59); Long-term exposure OR 0.97 (0.69–1.37)	No information on confounding factors; Prone to multiple testing
[129] Baumgardt-Elms et al.	Case-control	General population of five German cities (269 cases and 797 controls)	Self-reported and expert assessment	Testicular cancer	OR 1.0 (0.60–1.75)	Only 57% of identified controls participated
[148] Beard et al.	Case-control	U.S. military veterans (621 cases and 958 controls)	Self-reported	Amyotrophic lateral sclerosis	OR 1.74 (0.89–3.38)	Likely under-ascertainment of non-exposed cases; Prone to multiple testing
[125] Dabouis	Cohort (retrospective)	French Navy personnel followed from 1975 to 2000 (39,850 men)	Expert assessment	All-cause mortality All-cancer mortality	RR 1.0 (0.88–1.14) RR 0.92 (0.69–1.24)	43 % missing causes of death; No information on relevant confounding factors
[127] Davis and Mostofi	Cohort (retrospective)	Officers from two police departments in Washington, US, followed from 1979 to 1991 (340 men)	Job title	Testicular cancer	O/E 6.9 (*p* < 0.001)	Exposure was only assessed for the 6 cases in the cohort; No information on confounding factors
[145] De Roos et al.	Case-control	General US and Canadian population (538 cases and 504 controls, children)	Expert assessment	Neuroblastoma in offspring	OR 2.2 (0.6–8.3)	Result based on only 9 cases and 3 controls exposed to radar
[123] Degrave et al.	Cohort (retrospective)	Belgian military personnel followed from 1968 to 2003 (27,671 men)	Job title	All-cause mortality	SMR 1.05 (0.95–1.16)	Not all causes of death ascertained (76% in the radar group and 72% in the control group); No information on relevant confounding factors
[124] Degrave et al.	Cohort (retrospective)	Belgian professional military personnel followed from 1968–2004 (7,349 men)	Job title	All-cause mortality All-cancer mortality	RR 1.04 (0.96–1.14) RR 1.23 (1.03–1.47)	Not all causes of death ascertained (71% in the radar group and 70% in the control group); No information on relevant confounding factors
[137] Fabbro-Peray et al.	Case-control	General population of Languedoc-Roussillon, France (445 cases and 1025 controls)	Job title	Non-Hodgkin’s lymphoma	OR 1.3 (0.5–3.3)	Low participation among the controls (52.2%); Low number of cases (7) and controls (14) exposed to radar
[136] Finkelstein	Cohort (retrospective)	Officers from police departments in Ontario, Canada, followed from 1964 to 1995 (22,197 men)	Job title	All-cancer Melanoma	SIR 0.9 (0.83–0.98) SIR 1.45 (1.10–1.88)	No information on confounding factors; Significant loss to follow up (22%)
[131] Grayson	Case-control (nested)	US Air Force service personnel (230 cases and 920 controls, men)	Job-exposure matrix	Brain cancer	OR 1.39 (1.01–1.9)	Lack of diagnosis confirmation; No potential confounders were included in the analysis
[122] Groves et al.	Cohort (retrospective)	US Navy personnel followed from 1950 to 1997 (40,890 men)	Job title	All-cause mortality All-cancer mortality	RR 0.87 (0.83–0.9) RR 0.8 (0.74–0.87)	Under-ascertainment of cases; Limited exposure assessment period; No information on possible confounders
[128] Hardell et al.	Case-control	General Swedish population (148 cases and 314 controls, men)	Job title	Testicular cancer	OR 2.0 (0.3–14.2)	Result based on only 2 radar workers and 3 controls
[126] Hayes et al.	Case-control	Patients from medical institutions in Washington, US (271 cases and 259 controls, men)	Self-reported	Testicular cancer	OR 1.1 (0.7–1.9)	Short exposure period; No response rates reported
[133] Holly et al.	Case-control	Patients from the Ocular Oncology Unit at the University of California, US (221 cases of 447 controls, men)	Self-reported	Uveal melanoma	OR 2.1 (1.1–4.0)	Prone to multiple testing
[135] LA Vecchia et al.	Case-control	General population Milan, Italy (263 cases and 287 controls)	Self-reported	Bladder Cancer	No association, risk estimate not reported	Prone to multiple testing; Small number of cases across all the agents investigated
[146] Mageroy et al.	Cross-sectional	Norwegian Navy personnel (3,100 births from 1,438 parents)	Self-reported	Congenital anomalies	OR 4.0 (1.9–8.6)	The response rate was only 58%; Prone to multiple testing
[144] Mollerlokken et al.	Cross-sectional	Norwegian Navy personnel (3,752 men)	Expert assessment	Infertility	OR 2.28 (1.27–4.09)	No adjustment made for time spent on a boat
[121] Robinette et al.	Cohort study (retrospective)	US Navy enlisted personnel followed from 1950 to 1974 (40,890 men)	Job title	All-cause mortality All-cancer mortality	MR 0.96 MR 1.04	Under-ascertainment of cases; Limited exposure assessment period; No information on possible confounding factors
[132] Santana et al.	Case-control	Brazilian Navy personnel (40 cases and 671 controls, men)	Job title	Brain cancer	OR 0.56 (0.17–1.82)	Small number of cases (40); Lack of diagnosis confirmation; Use of last job title only
[134] Stang et al.	Case-control	General population of Essen, Germany (118 cases and 475 controls)	Self-reported	Uveal Melanoma	OR 0.4 (0.0–2.6)	High non-response among the population controls (52%)
[138] Variani	Meta-analysis	Populations from Groves et al. (2002), Degrave et al. (2009) and Dabouis et al. (2014)	Various	All-cancer mortality	MR 0.81 (0.78–0.83)	Only six studies included in the meta-analysis with significant heterogeneity between studies
[142] Velez de la Calle et al.	Case-control	Military personnel from Brest, France (60 cases and 165 controls, couples)	Self-reported	Infertility	OR 0.8 (0.4–1.6)	No comparison in sperm characteristics between cases and controls
[130] Walschaerts	Case-control	Patients from 5 cities in France (229 cases and 800 controls, men)	Job title	Testicular cancer	OR 0.84 (0.38–1.87)	Low participation (39%) in control group

*OR* Odds ratio, *RR* Relative risk, *O/E* Observed to expected ratio, *SIR* Standardised incidence ratio, *MR* Mortality ratio

Three large cohort studies investigated mortality in military personnel with potential exposure to MMWs from radar. Studies reporting on over 40-year follow-up of US navy veterans of the Korean War found that radar exposure had little effect on all-cause or cancer mortality with the second study reporting risk estimates below unity [121, 122]. Similarly, in a 40-year follow-up of Belgian military radar operators, there was no statistically significant increase in all-cause mortality [123, 124]; the study did, however, find a small increase in cancer mortality. More recently in a 25-year follow-up of military personnel who served in the French Navy, there was no increase in all-cause or cancer mortality for personnel exposed to radar [125]. The main limitation in the cohort studies was the lack of individual levels of RF exposure with most studies based on job-title. Comparisons were made between occupations with presumed high exposure to RF fields and other occupations with presumed lower exposure. This type of non-differential misclassification in dichotomous exposure assessment is associated mostly with an effect measure biased towards a null effect if there is a true effect of RF fields. If there is no true effect of RF fields, non-differential exposure misclassification will not bias the effect estimate (which will be close to the null value, but may vary because of random error). The military personnel in these studies were compared with the general population and this ‘healthy worker effect’ presents possible bias since military personnel are on average in better health than the general population; the healthy worker effect tends to underestimate the risk. The cohort studies also lacked information on possible confounding factors including other occupational exposures such as chemicals and lifestyle factors such as smoking.

Several epidemiological studies have specifically investigated radar exposure and testicular cancer. In a case-control study where most of the subjects were selected from military hospitals in Washington DC, USA, Hayes et al. found no increased risk between exposure to radar and testicular cancer [126]; exposure to radar was self-reported and thus subject to misclassification. In this study, the misclassification was likely non-differential, biasing the result towards the null. Davis and Mostofi reported a cluster of testicular cancer within a small cohort of 340 police officers in Washington State (USA) where the cases routinely used handheld traffic radar guns [127]; however, exposure was not assessed for the full cohort, which may have overestimated the risk. In a population-based case-control study conducted in Sweden, Hardell et al. did not find a statistically significant association between radar work and testicular cancer; however, the result was based on only five radar workers questioning the validity of this result [128]. In a larger population-based case control study in Germany, Baumgardt-Elms et al. also reported no association between working near radar units (both self-reported and expert assessed) and testicular cancer [129]; a limitation of this study was the low participation of identified controls (57%), however, there was no difference compared with the characteristics of the cases so selection bias was unlikely. In the cohort study of US navy veterans previously mentioned exposure to radar was not associated with testicular cancer [122]; the limitations of this cohort study mentioned earlier may have underestimated the risk. Finally, in a hospital-based case-control study in France, radar workers were also not associated with risk of testicular cancer [130]; a limitation was the low participation of controls (37%) with a difference in education level between participating and non-participating controls, which may have underestimated this result.

A limited number of studies have investigated radar exposure and brain cancer. In a nested case-control study within a cohort of male US Air Force personnel, Grayson reported a small association between brain cancer and RF exposure, which included radar [131]; no potential confounders were included in the analysis, which may have overestimated the result. However, in a case-control study of personnel in the Brazilian Navy, Santana et al. reported no association between naval occupations likely to be exposed to radar and brain cancer [132]; the small number of cases and lack of diagnosis confirmation may have biased the results towards the null. All of the cohort studies on military personnel previously mentioned also examined brain cancer mortality and found no association with exposure to radar [122, 124, 125].

A limited number of studies have investigated radar exposure and ocular cancer. Holly et al. in a population-based case-control study in the US reported an association between self-reported exposure to radar or microwaves and uveal melanoma [133]; the study investigated many different exposures and the result is prone to multiple testing. In another case-control study, which used both hospital and population controls, Stang et al. did not find an association between self-reported exposure to radar and uveal melanoma [134]; a high non-response in the population controls (52%) and exposure misclassification may have underestimated this result. The cohort studies of the Belgian military and French navy also found no association between exposure to radar and ocular cancer [124, 125].

A few other studies have examined the potential association between radar and other cancers. In a hospital-based case-control study in Italy, La Vecchia investigated 14 occupational agents and risk of bladder cancer and found no association with radar, although no risk estimate was reported [135]; non-differential self-reporting of exposure may have underestimated this finding if there is a true effect. Finkelstein found an increased risk for melanoma in a large cohort of Ontario police officers exposed to traffic radar and followed for 31 years [136]; there was significant loss to follow up which may have biased this result in either direction. Finkelstein found no statistically significant associations with other types of cancer and the study reported a statistically significant risk estimate just below unity for all cancers, which is reflective of the healthy worker effect [136]. In a large population-based case-control study in France, Fabbro-Peray et al. investigated a large number of occupational and environmental risk factors in relation to non-Hodgkin lymphoma and found no association with radar operators based on job-title; however, the result was based on a small number of radar operators [137]. The cohort studies on military personnel did not find statistically significant associations between exposure to radar and other cancers [122, 124, 125].

Variani et al. conducted a recent systematic review and meta-analysis investigating occupational exposure to radar and cancer risk [138]. The meta-analysis included three cohort studies [122, 124, 125] and three case-control studies [129–131] for a total sample size of 53,000 subjects. The meta-analysis reported a decrease in cancer risk for workers exposed to radar but noted the small number of studies included with significant heterogeneity between the studies.

Apart from cancer, a number of epidemiological studies have investigated radar exposure and reproductive outcomes. Two early studies on military personnel in the US [139] and Denmark [140] reported differences in semen parameters between personnel using radar and personnel on other duty assignments; these studies included only volunteers with potential fertility concerns and are prone to bias. A further volunteer study on US military personnel did not find a difference in semen parameters in a similar comparison [141]; in general these type of cross-sectional investigations on volunteers provide limited evidence on possible risk. In a case-control study of personnel in the French military, Velez de la Calle et al. reported no association between exposure to radar and male infertility [142]; non-differential self-reporting of exposure may have underestimated this finding if there is a true effect. In two separate cross-sectional studies of personnel in the Norwegian navy, Baste et al. and Møllerløkken et al. reported an association between exposure to radar and male infertility, but there has been no follow up cohort or case control studies to confirm these results [143, 144].

Again considering reproduction, a number of studies investigated pregnancy and offspring outcomes. In a population-based case-control study conducted in the US and Canada, De Roos et al. found no statistically significant association between parental occupational exposure to radar and neuroblastoma in offspring; however, the result was based on a small number of cases and controls exposed to radar [145]. In another cross-sectional study of the Norwegian navy, Mageroy et al. reported a higher risk of congenital anomalies in the offspring of personnel who were exposed to radar; the study found positive associations with a large number of other chemical and physical exposures, but the study involved multiple comparisons so is prone to over-interpretation [146]. Finally, a number of pregnancy outcomes were investigated in a cohort study of Norwegian navy personnel enlisted between 1950 and 2004 [147]. The study reported an increase in perinatal mortality for parental service aboard fast patrol boats during a short period (3 months); exposure to radar was one of many possible exposures when serving on fast patrol boats and the result is prone to multiple testing. No associations were found between long-term exposure and any pregnancy outcomes.

There is limited research investigating exposure to radar and other diseases. In a large case-control study of US military veterans investigating a range of risk factors and amyotrophic lateral sclerosis, Beard et al. did not find a statistically significant association with radar [148]; the study reported a likely under-ascertainment of non-exposed cases, which may have biased the result away from the null. The cohort studies on military personnel did not find statistically significant associations between exposure to radar and other diseases [122, 124, 125].

A number of observational studies have investigated outcomes measured on volunteers in the laboratory. They are categorised as epidemiological studies because exposure to radar was not based on provocation. These studies investigated genotoxicity [149], oxidative stress [149], cognitive effects [150] and endocrine function [151]; the studies generally reported positive associations with radar. These volunteer studies did not sample from a defined population and are prone to bias [152].

## Discussion

The experimental studies investigating exposure to MMWs at levels below the ICNIRP occupational limits have looked at a variety of biological effects. Genotoxicity was mainly examined by using comet assays of exposed cells. This approach has consistently found no evidence of DNA damage in skin cells in well-designed studies. However, animal studies conducted by one research group reported DNA strand breaks and changes in enzymes that control the build-up of ROS, noting that these studies had low animal numbers (six animals exposed); these results have not been independently replicated. Studies have also investigated other indications of genotoxicity including chromosome aberrations, micro-nucleation and spindle disturbances. The methods used to investigate these indicators have generally been rigorous; however, the studies have reported contradictory results. Two studies by a Russian research group have also reported indicators of DNA damage in bacteria, however, these results have not been verified by other investigators.

The studies of the effect of MMWs on cell proliferation primarily focused on bacteria, yeast cells and tumour cells. Studies of bacteria were mainly from an Armenian research group that reported a reduction in the bacterial growth rate of exposed E. coli cells at different MMW frequencies; however, the studies suffered from inadequate dosimetry and temperature control and heating due to high RF energy deposition may have contributed to the results. Other authors have reported no effect of MMWs on E. coli cell growth rate. The results on cell proliferation of yeast exposed to MMWs were also contradictory. An Italian research group that has conducted the majority of the studies on tumour cells reported either a reduction or no change in the proliferation of exposed cells; however, these studies also suffered from inadequate dosimetry and temperature control.

The studies on gene expression mainly examined two different indicators, expression of stress sensitive genes and chaperone proteins and the occurrence of a resonance effect in cells to explain DNA conformation state changes. Most studies reported no effect of low-level MMWs on the expression of stress sensitive genes or chaperone proteins using a range of experimental methods to confirm these results; noting that these studies did not use blinding so experimental bias cannot be excluded from the results. A number of studies from a Russian research group reported a resonance effect of MMWs, which they propose can change the conformation state of chromosomal DNA complexes. Their results relied heavily on the AVTD method for testing changes in the DNA conformation state, however, the biological relevance of results obtained through the AVTD method has not been independently validated.

Studies on cell signalling and electrical activity reported a range of different outcomes including increases or decreases in signal amplitude and changes in signal rhythm, with no consistent effect noting the lack of blinding in most of the studies. Further, temperature contributions could not be eliminated from the studies and in some cases thermal interactions by conventional heating were studied and found to differ from the MMW effects. The results from some studies were based on small sample sizes, some being confined to a single specimen, or by observed effects only occurring in a small number of the samples tested. Overall, the reported electrical activity effects could not be dismissed as being within normal variability. This is indicated by studies reporting the restoration of normal function within a short time during ongoing exposure. In this case there is no implication of an expected negative health outcome.

Studies on membrane effects examined changes in membrane properties and permeability. Some studies observed changes in transitions from liquid to gel phase or vice versa and the authors implied that MMWs influenced cell hydration, however the statistical methods used in these studies were not described so it is difficult to examine the validity of these results. Other studies observing membrane properties in artificial cell suspensions and dissected tissue reported changes in vesicle shape, reduced cell volume and morphological changes although most of these studies suffered from various methodological problems including poor temperature control and no blinding. Experiments on bacteria and yeast were conducted by the same research group reporting changes in membrane permeability, which was attributed to cell proliferation effects, however, the studies suffered from inadequate dosimetry and temperature control. Overall, although there were a variety of membrane bioeffects reported, these have not been independently replicated.

The limited number of studies on a number of other effects from exposure to MMWs below the ICNIRP limits generally reported little to no consistent effects. The single in vivo study on cancer promotion did not find an effect although the study did not include sham controls. Effects on reproduction were contradictory that may have been influenced by opposing objectives of examining adverse health effects or infertility treatment. Further, the only study on human sperm found no effects of low-level MMWs. The studies on reproduction suffered from inadequate dosimetry and temperature control, and since sperm is sensitive to temperature, the effect of heating due to high RF energy deposition may have contributed to the studies showing an effect. A number of studies from two research groups reported effects on ROS production in relation to reproduction and immune function; the in vivo studies had low animal numbers (six animals per exposure) and the in vitro studies generally had inadequate dosimetry and temperature control. Studies on fatty acid composition and physiological indicators did not generally show any effects; poor temperature control was also a problem in the majority of these studies. A number of other studies investigating various other biological effects reported mixed results.

Although a range of bioeffects have been reported in many of the experimental studies, the results were generally not independently reproduced. Approximately half of the studies were from just five laboratories and several studies represented a collaboration between one or more laboratories. The exposure characteristics varied considerably among the different studies with studies showing the highest effect size clustered around a PD of approximately 1 W/m^2^. The meta-analysis of the experimental studies in our companion paper [9] showed that there was no dose-response relationship between the exposure (either PD or SAR) and the effect size. In fact, studies with a higher exposure tended to show a lower effect size, which is counterfactual. Most of the studies showing a large effect size were conducted in the frequency range around 40–55 GHz, representing investigations into the use of MMWs for therapeutic purposes, rather than deleterious health consequences. Future experimental research would benefit from investigating bioeffects at the specific frequency range of the next stage of the 5 G network roll-out in the range 26–28 GHz. Mobile communications beyond the 5 G network plan to use frequencies higher than 30 GHz so research across the MMW band is relevant.

An investigation into the methods of the experimental studies showed that the majority of studies were lacking in a number of quality criteria including proper attention to dosimetry, incorporating positive controls, using blind evaluation or accurately measuring or controlling the temperature of the biological system being tested. Our meta-analysis showed that the bulk of the studies had a quality score lower than 2 out of a possible 5, with only one study achieving a maximum quality score of 5 [9]. The meta-analysis further showed that studies with a low quality score were more likely to show a greater effect. Future research should pay careful attention to the experimental design to reduce possible sources of artefact.

The experimental studies included in this review reported PDs below the ICNIRP exposure limits. Many of the authors suggested that the resulting biological effects may be related to non-thermal mechanisms. However, as is shown in our meta-analysis, data from these studies should be treated with caution because the estimated SAR values in many of the studies were much higher than the ICNIRP SAR limits [9]. SAR values much higher than the ICNIRP guidelines are certainly capable of producing significant temperature rise and are far beyond the levels expected for 5 G telecommunication devices [1]. Future research into the low-level effects of MMWs should pay particular attention to appropriate temperature control in order to avoid possible heating effects.

Although a systematic review of experimental studies was not conducted, this paper presents a critical appraisal of study design and quality of all available studies into the bioeffects of low level MMWs. The conclusions from the review of experimental studies are supported by a meta-analysis in our companion paper [9]. Given the low-quality methods of the majority of the experimental studies we infer that a systematic review of different bioeffects is not possible at present. Our review includes recommendations for future experimental research. A search of the available literature showed a further 44 non-English papers that were not included in our review. Although the non-English papers may have some important results it is noted that the majority are from research groups that have published English papers that are included in our review.

The epidemiological studies on MMW exposure from radar that has a similar frequency range to that of 5 G and exposure levels below the ICNIRP occupational limits in most situations, provided little evidence of an association with any adverse health effects. Only a small number of studies reported positive associations with various methodological issues such as risk of bias, confounding and multiple testing questioning the result. The three large cohort studies of military personnel exposed to radar in particular did not generally show an association with cancer or other diseases. A key concern across all the epidemiological studies was the quality of exposure assessment. Various challenges such as variability in complex occupational environments that also include other co-exposures, retrospective estimation of exposure and an appropriate exposure metric remain central in studies of this nature [153]. Exposure in most of the epidemiological studies was self-reported or based on job-title, which may not necessarily be an adequate proxy for exposure to RF fields above 6 GHz. Some studies improved on exposure assessment by using expert assessment and job-exposure matrices, however, the possibility of exposure misclassification is not eliminated. Another limitation in many of the studies was the poor assessment of possible confounding including other occupational exposures and lifestyle factors. It should also be noted that close proximity to certain very powerful radar units could have exceeded the ICNIRP occupational limits, therefore the reported effects especially related to reproductive outcomes could potentially be related to heating.

Given that wireless communications have only recently started to use RF frequencies above 6 GHz there are no epidemiological studies investigating 5 G directly as yet. Some previous epidemiological studies have reported a possible weak association between mobile phone use (from older networks using frequencies below 6 GHz) and brain cancer [11]. However, methodological limitations in these studies prevent conclusions of causality being drawn from the observations [152]. Recent investigations have not shown an increase in the incidence of brain cancer in the population that can be attributed to mobile phone use [154, 155]. Future epidemiological research should continue to monitor long-term health effects in the population related to wireless telecommunications.

The review of experimental studies provided no confirmed evidence that low-level MMWs are associated with biological effects relevant to human health. Many of the studies reporting effects came from the same research groups and the results have not been independently reproduced. The majority of the studies employed low quality methods of exposure assessment and control so the possibility of experimental artefact cannot be excluded. Further, many of the effects reported may have been related to heating from high RF energy deposition so the assertion of a ‘low-level’ effect is questionable in many of the studies. Future studies into the low-level effects of MMWs should improve the experimental design with particular attention to dosimetry and temperature control. The results from epidemiological studies presented little evidence of an association between low-level MMWs and any adverse health effects. Future epidemiological research would benefit from specific investigation on the impact of 5 G and future telecommunication technologies.

## References

[1] Wu T, Rappaport TS, Collins CM (2015). Safe for generations to come: considerations of safety for millimeter waves in wireless communications. IEEE Micro Mag.

[2] Health protection agency (HPA). Health effects from radiofrequency electromagnetic fields: the report of the independent advisory group on non-ionising radiation (AGNIR). HPA. 2012; RCE 20.

[3] Scientific committee on emerging and newly identified health risks (SCENHIR). Potential health effects of exposure to electromagnetic fields (EMF). Euro Comm. 2015; 1831-4783.

[4] Australian radiation protection and nuclear safety agency (ARPANSA). Radiation protection standard for maximum exposure levels to radiofrequency fields—3 kHz to 300 GHz. Radiation Protection Series 3. ARPANSA; 2002.

[5] International Commission on Non-Ionizing Radiation Protection (ICNIRP). (2020). ICNIRP guidelines for limiting exposure to electromagnetic fields (100 KHz to 300 GHz). Health Phys.

[6] Institute of electrical and electronics engineers (IEEE). IEEE standard for safety levels with respect to human exposure to electric, magnetic, and electromagnetic fields, 0 Hz to 300 GHz. IEEE 2019; C95.1.

[7] Stam R. Comparison of international policies on electromagnetic fields (power frequency and radiofrequency fields). National institute for public health and the environment, RIVM 2018.

[8] Simkó M, Mattsson MO (2019). 5G Wireless communication and health effects—a pragmatic review based on available studies regarding 6 to 100 GHz. Int J Environ Res Public Health.

[9] Wood A, Mate R, Karipidis K. Meta-analysis of in vitro and in vivo studies of the biological effects of low-level millimetre waves. 2020. 10.1038/s41370-021-00307-7. 10.1038/s41370-021-00307-7 PMC7962924 33727686

[10] International commission on non-Ionizing radiation protection (ICNIRP). Exposure to high frequency electromagnetic fields, biological effects and health consequences (100 kHz-300 GHz). ICNIRP 2009; 978-3-934994-10-2.

[11] International agency for research on cancer (IARC) (2013). IARC monographs: non-ionizing radiation, part 2: radiofrequency electromagnetic fields. IARC.

[12] Garaj-Vrhovac V, Horvat D, Koren Z (1991). The relationship between colony-forming ability, chromosome aberrations and incidence of micronuclei in V79 Chinese hamster cells exposed to microwave radiation. Mutat Res Lett.

[13] Garaj-Vrhovac V, Fučić A, Horvat D (1992). The correlation between the frequency of micronuclei and specific chromosome aberrations in human lymphocytes exposed to microwave radiation in vitro. Mutat Res Lett.

[14] Korenstein-Ilan A, Barbul A, Hasin P, Eliran A, Gover A, Korenstein R (2008). Terahertz radiation increases genomic instability in human lymphocytes. Radiat Res.

[15] Hintzsche H Jastrow C, Kleine-Ostmann T, Kärst U, Schrader T, Stopper H (2012). Terahertz electromagnetic fields (0.106 THz) do not induce manifest genomic damage in vitro. PloS One.

[16] Koyama S, Narita E, Shimizu Y, Suzuki Y, Shiina T, Taki M (2016). Effects of long-term exposure to 60 GHz millimeter-wavelength radiation on the genotoxicity and heat shock protein (Hsp) expression of cells derived from human eye. Int J Environ Res Public Health.

[17] Koyama S, Narita E, Suzuki Y, Shiina T, Taki M, Shinohara N (2019). Long-term exposure to a 40-GHz electromagnetic field does not affect genotoxicity or heat shock protein expression in HCE-T or SRA01/04 cells. J Radiat Res.

[18] De Amicis A, De Sanctis S, Di Cristofaro S, Franchini V, Lista F, Regalbuto E (2015). Biological effects of in vitro THz radiation exposure in human foetal fibroblasts. Mutat Res Genet Toxicol Environ Mutagen.

[19] Franchini V, Regalbuto E, De Amicis A, De Sanctis S, Di Cristofaro S, Coluzzi E (2018). Genotoxic effects in human fibroblasts exposed to microwave radiation. Health Phys.

[20] Shckorbatov YG, Grigoryeva NN, Shakhbazov VG, Grabina VA, Bogoslavsky AM (1998). Microwave irradiation influences on the state of human cell nuclei. Bioelectromagnetics.

[21] Shckorbatov YG, Pasiuga VN, Kolchigin NN, Grabina VA, Batrakov DO, Kalashnikov VV (2009). The influence of differently polarised microwave radiation on chromatin in human cells. Int J Radiat Biol.

[22] Shckorbatov YG, Pasiuga VN, Goncharuk EI, Petrenko TP, Grabina VA, Kolchigin NN (2010). Effects of differently polarized microwave radiation on the microscopic structure of the nuclei in human fibroblasts. J Zhejiang Univ Sci B.

[23] Paulraj R, Behari J (2006). Single strand DNA breaks in rat brain cells exposed to microwave radiation. Mutat Res.

[24] Kesari KK, Behari J (2009). Fifty-gigahertz microwave exposure effect of radiations on rat brain. Appl Biochem Biotechnol.

[25] Kumar S, Kesari KK, Behari J (2010). Evaluation of genotoxic effects in male Wistar rats following microwave exposure. Indian J Exp Biol.

[26] Crouzier D, Perrin A, Torres G, Dabouis V, Debouzy JC (2009). Pulsed electromagnetic field at 9.71 GHz increase free radical production in yeast (Saccharomyces cerevisiae). Patho Biol.

[27] Smolyanskaya AZ, Vilenskaya RL (1974). Effects of millimeter-band electromagnetic radiation on the functional activity of certain genetic elements of bacterial cells. Sov Phys.

[28] Lukashevsky KV, Belyaev IY (1990). Switching of prophage lambda genes in Escherichia coli by millimetre waves. Med Sci Res.

[29] Kalantaryan VP, Vardevanyan PO, Babayan YS, Gevorgyan ES, Hakobyan SN, Antonyan AP (2010). Influence of low intensity coherent electromagnetic millimeter radiation (EMR) on aqua solution of DNA. Prog Electromag Res.

[30] Hintzsche H, Jastrow C, Kleine-Ostmann T (2011). Terahertz radiation induces spindle disturbances in human-hamster hybrid cells. Radiat Res.

[31] Zeni O, Gallerano GP, Perrotta A, Romano M, Sannino A, Sarti M (2007). Cytogenetic observations in human peripheral blood leukocytes following in vitro exposure to THz radiation: a pilot study. Health Phys.

[32] Gapeyev A, Lukyanova N, Gudkov S (2014). Hydrogen peroxide induced by modulated electromagnetic radiation protects the cells from DNA damage. Open Life Sci.

[33] Gapeyev AB, Lukyanova NA (2015). Pulse-modulated extremely high-frequency electromagnetic radiation protects cellular DNA from the damaging effects of physical and chemical factors in vitro. Biophys.

[34] Webb SJ, Dodds DD (1968). Inhibition of bacterial cell growth by 136 GC microwaves. Nature.

[35] Webb SJ, Booth AD (1969). Absorption of microwaves by microorganisms. Nature.

[36] Rojavin MA, Ziskin MC (1995). Effect of millimeter waves on survival of UVC‐exposed Escherichia coli. Bioelectromagnetics.

[37] Pakhomova ON, Pakhomov AG, Akyel Y (1997). Effect of millimeter waves on UV-induced recombination and mutagenesis in yeast. Bioelectrochem Bioenerg.

[38] Cohen I, Cahan R, Shani G, Cohen E, Abramovich A (2010). Effect of 99 GHz continuous millimeter wave electro-magnetic radiation on E. coli viability and metabolic activity. Int J Radiat Biol.

[39] Tadevosyan H, Kalantaryan V, Trchounian A (2008). Extremely high frequency electromagnetic radiation enforces bacterial effects of inhibitors and antibiotics. Cell Biochem Biophys.

[40] Torgomyan H, Trchounian A (2011). Low-intensity electromagnetic irradiation of 70.6 and 73 GHz frequencies enhances the effects of disulfide bonds reducer on Escherichia coli growth and affects the bacterial surface oxidation–reduction state. Biochem Biophys Res Commun.

[41] Torgomyan H, Kalantaryan V, Trchounian A (2011). Low intensity electromagnetic irradiation with 70.6 and 73 GHz frequencies affects Escherichia coli growth and changes water properties. Cell Biochem Biophys.

[42] Torgomyan H, Hovnanyan K, Trchounian A (2012). Escherichia coli growth changes by the mediated effects after low-intensity electromagnetic irradiation of extremely high frequencies. Cell Biochem Biophys.

[43] Torgomyan H, Ohanyan V, Blbulyan S, Kalantaryan V, Trchounian A (2012). Electromagnetic irradiation of Enterococcus hirae at low-intensity 51.8-and 53.0-GHz frequencies: changes in bacterial cell membrane properties and enhanced antibiotics effects. FEMS microbiol Lett.

[44] Soghomonyan D, Trchounian A (2013). Comparable effects of low-intensity electromagnetic irradiation at the frequency of 51.8 and 53 GHz and antibiotic ceftazidime on Lactobacillus acidophilus growth and survival. Cell Biochem Biophys.

[45] Hovnanyan K, Kalantaryan V, Trchounian A (2017). The distinguishing effects of low‐intensity electromagnetic radiation of different extremely high frequencies on Enterococcus hirae: growth rate inhibition and scanning electron microscopy analysis. Lett Appl microbiol.

[46] Grundler W, Keilmann F (1977). Nonthermal effects of millimeter microwaves on yeast growth. Z Naturforsch.

[47] Grundler W, Keilmann F (1983). Sharp resonances in yeast growth prove nonthermal sensitivity to microwaves. Phys Rev Lett.

[48] Furia L, Hill DW, Gandhi OMP (1986). Effect of millimeter-wave irradiation on growth of Saccharomyces cerevisiae. IEEE Trans Biom Eng.

[49] Gos P, Eicher B, Kohli J, Heyer WD (1997). Extremely high frequency electromagnetic fields at low power density do not affect the division of exponential phase Saccharomyces cerevisiae cells. Bioelectromagnetics.

[50] Chidichimo G, Beneduci A, Nicoletta M, Critelli M, De RR, Tkatchenko Y (2002). Selective inhibition of tumoral cells growth by low power millimeter waves. Anticancer Res.

[51] Beneduci A, Chidichimo G, Tripepi S, Perrotte E (2005). Frequency and irradiation time-dependant antiproliferative effect of low-power millimeter waves on RPMI 7932 human melanoma cell line. Anticancer Res.

[52] Beneduci A, Chidichimo G, Tripepi S, Perrotte E (2005). Transmission electron microscopy study of the effects produced by wide-band low-power millimeter waves on MCF-7 human breast cancer cells in culture. Anticancer Res.

[53] Beneduci A (2009). Evaluation of the potential in vitro antiproliferative effects of millimeter waves at some therapeutic frequencies on RPMI 7932 human skin malignant melanoma cells. Cell Biochem Biophys.

[54] Beneduci A, Chidichimo G, Tripepi S, Perrotta E, Cufone F (2007). Antiproliferative effect of millimeter radiation on human erythromyeloid leukemia cell line K562 in culture: ultrastructural-and metabolic-induced changes. Bioelectrochemistry.

[55] Yaekashiwa N, Otsuki S, Hayashi SI, Kawase K (2017). Investigation of the non-thermal effects of exposing cells to 70–300 GHz irradiation using a widely tunable source. J Radiat Res.

[56] Badzhinyan SA, Sayadyan AB, Sarkisyan NK, Grigoryan RM, Gasparyan GG (2001). Lethal effect of electromagnetic radiation of the millimeter wavelength range on cell cultures of chicken embryo. Dokl Biochem Biophys.

[57] Shiina T, Suzuki Y, Kasai Y, Inami Y, Taki M, Wake K (2014). Effect of two-times 24 h exposures to 60 GHz millimeter-waves on neurite outgrowth in PC12VG cells in consideration of polarization. IEEE Int Sympo Electromag Compat.

[58] Le Quément C, Nicolas Nicolaz C, Zhadobov M, Desmots F, Sauleau R, Aubry M (2012). Whole‐genome expression analysis in primary human keratinocyte cell cultures exposed to 60 GHz radiation. Bioelectromagnetics.

[59] Zhadobov M, Sauleau R, Le Coq L, Thouroude D, Orlov I, Michel D (2005). 60 GHz electromagnetic fields do not activate stress-sensitive gene expression. IEEE 11th Int Sympo on Antenna Technol and appl electromag.

[60] Zhadobov M, Sauleau R, Le Coq L, Debure L, Thouroude D, Michel D (2007). Low‐power millimeter wave radiations do not alter stress‐sensitive gene expression of chaperone proteins. Bioelectromagnetics.

[61] Zhadobov M, Nicolaz CN, Sauleau R, Desmots F, Thouroude D, Michel D (2009). Evaluation of the potential biological effects of the 60-GHz millimeter waves upon human cells. IEEE Trans Antennas Propag.

[62] Nicolaz CN, Zhadobov M, Desmots F, Ansart A, Sauleau R, Thouroude D (2008). Study of narrow band millimeter‐wave potential interactions with endoplasmic reticulum stress sensor genes. Bioelectromagnetics.

[63] Nicolaz CN, Zhadobov M, Desmots F, Sauleau R, Thouroude D, Michel D (2009). Absence of direct effect of low-power millimeter-wave radiation at 60.4 GHz on endoplasmic reticulum stress. Cell Biol Toxicol.

[64] Belyaev IY, Alipov YD, Shcheglov VS, Lystsov VN (1992). Resonance effect of microwaves on the genome conformational state of E. coli cells. Z Naturforsch C.

[65] Belyaev IY, Shcheglov VS, Alipov YD (1992). Existence of selection rules on helicity during discrete transitions of the genome conformational state of E. coli cells exposed to low-level millimetre radiation. Bioelectrochem Bioenerg.

[66] Belyaev IY, Shcheglov VS, Alipov YD (1992). Selection rules on helicity during discrete transitions of the genome conformational state in intact and X-rayed cells of E. coli in millimeter range of electromagnetic field. Charg Field Eff Biosyst.

[67] Belyaev I, Alipov YD, Shcheglov VS, Chromosome DNA (1992). as a target of resonant interaction between Escherichia coli cells and low–intensity millimeter waves. Electro Magnetobiol.

[68] Belyaev IY, Alipov YD, Polunin VA, Shcheglov VS (1993). Evidence for dependence of resonant frequency of millimeter wave interaction with Escherichia coli K12 cells on haploid genome length. Electro Magnetobiol.

[69] Belyaev IY, Shcheglov VS, Alipov YD, Radko SP (1993). Regularities of separate and combined effects of circularly polarized millimeter waves on E. coli cells at different phases of culture growth. Bioelectrochem Bioenerg.

[70] Belyaev IY, Alipov YD, Shcheglov VS, Polunin VA, Aizenberg OA (1994). Cooperative response of Escherichia coli cells to the resonance effect of millimeter waves at super low intensity. Electro Magnetobiol.

[71] Belyaev IY, Kravchenko VG (1994). Resonance effect of low-intensity millimeter waves on the chromatin conformational state of rat thymocytes. Z Naturforsch.

[72] Belyaev IY, Shcheglov VS, Alipov YD, Polunin VA (1996). Resonance effect of millimeter waves in the power range from 10‐19 to 3× 10‐3 W/cm2 on Escherichia coli cells at different concentrations. Bioelectromagnetics.

[73] Shcheglov VS, Belyaev I, Alipov YD, Ushakov VL (1997). Power-dependent rearrangement in the spectrum of resonance effect of millimeter waves on the genome conformational state of Escherichia Coli cells. Electro Magnetobiol.

[74] Shcheglov VS, Alipov ED, Belyaev I (2002). Cell-to-cell communication in response of E. coli cells at different phases of growth to low-intensity microwaves. Biochim biophys Acta.

[75] Gandhi OP, Hagmann MJ, Hill DW, Partlow LM, Bush L (1980). Millimeter wave absorption spectra of biological samples. Bioelectromagnetics.

[76] Bush LG, Hill DW, Riazi A, Stensaas LJ, Partlow LM, Gandhi OP (1981). Effects of millimeter‐wave radiation on monolayer cell cultures. III. A search for frequency‐specific athermal biological effects on protein synthesis. Bioelectromagnetics.

[77] Belyaev IY, Shcheglov VS, Alipov ED, Ushakov VD (2000). Nonthermal effects of extremely high-frequency microwaves on chromatin conformation in cells in vitro—dependence on physical, physiological, and genetic factors. IEEE Trans Micro Theory Tech.

[78] Pakhomov AG, Akyel Y, Pakhomova ON, Stuck BE, Murphy MR (1998). Current state and implications of research on biological effects of millimeter waves: a review of the literature. Bioelectromagnetics.

[79] Minasyan SM, Grigoryan GY, Saakyan SG, Akhumyan AA, Kalantaryan VP (2007). Effects of the action of microwave-frequency electromagnetic radiation on the spike activity of neurons in the supraoptic nucleus of the hypothalamus in rats. Neurosci Behav Physiol.

[80] Pikov V, Arakaki X, Harrington M, Fraser SE, Siegel PH (2010). Modulation of neuronal activity and plasma membrane properties with low-power millimeter waves in organotypic cortical slices. J Neural Eng.

[81] Munemori J, Ikeda T (1982). Effects of low-level microwave radiation on the eye of the crayfish. Med Biol Eng Comput.

[82] Munemori J, Ikeda T (1984). Biological effects of X-band microwave radiation on the eye of the crayfish. Med Biol Eng Comput.

[83] Pakhomov AG, Prol HK, Mathur SP, Akyel Y, Campbell CB (1997). Frequency-specific effects of millimeter-wavelength electromagnetic radiation in isolated nerve. Electro Magnetobiol.

[84] Pakhomov AG, Prol HK, Mathur SP, Akyel Y, Campbell CB (1997). Search for frequency‐specific effects of millimeter‐wave radiation on isolated nerve function. Bioelectromagnetics.

[85] Pakhomov AG, Prol HK, Mathur SP, Akyel Y, Campbell CB (1997). Role of field intensity in the biological effectiveness of millimeter waves at a resonance frequency. Bioelectrochem Bioenerg.

[86] Pikov V, Siegel PH (2011). Millimeter wave-induced changes in membrane properties of leech Retzius neurons. Photonic Therapeutics Diagnostics.

[87] Romanenko S, Siegel PH, Pikov V (2013). Microdosimetry and physiological effects of millimeter wave irradiation in isolated neural ganglion preparation. IEEE 2013 International kharkov symposium on physics and engineering of microwaves, millimeter and submillimeter waves. IEEE.

[88] Romanenko S, Siegel PH, Wagenaar DA, Pikov V (2014). Effects of millimeter wave irradiation and equivalent thermal heating on the activity of individual neurons in the leech ganglion. J Neurophysiol.

[89] Beneduci A, Filippelli L, Cosentino K, Calabrese ML, Massa R, Chidichimo G (2012). Microwave induced shift of the main phase transition in phosphatidylcholine membranes. Bioelectrochemistry.

[90] Beneduci A, Cosentino K, Chidichimo G (2013). Millimeter wave radiations affect membrane hydration in phosphatidylcholine vesicles. Materials.

[91] Beneduci A, Cosentino K, Romeo S, Massa R, Chidichimo G (2014). Effect of millimetre waves on phosphatidylcholine membrane models: a non-thermal mechanism of interaction. Soft Matter.

[92] Geletyuk VI, Kazachenko VN, Chemeris NK, Fesenko EE (1995). Dual effects of microwaves on single Ca2+-activated K+ channels in cultured kidney cells Vero. FEBS Lett.

[93] Chen Q, Zeng QL, Lu DQ, Chiang H (2004). Millimeter wave exposure reverses TPA suppression of gap junction intercellular communication in HaCaT human keratinocytes. Bioelectromagnetics.

[94] Shckorbatov YG, Shakhbazov VG, Navrotskaya VV, Grabina VA, Sirenko SP, Fisun AI (2002). Application of intracellular microelectrophoresis to analysis of the influence of the low‐level microwave radiation on electrokinetic properties of nuclei in human epithelial cells. Electrophoresis.

[95] Zhadobov M, Sauleau R, Vié V, Himdi M, Le Coq L, Thouroude D (2006). Interactions between 60-GHz millimeter waves and artificial biological membranes: dependence on radiation parameters. IEEE Trans Micro Theory Tech.

[96] Deghoyan A, Heqimyan A, Nikoghosyan A, Dadasyan E, Ayrapetyan S (2012). Cell bathing medium as a target for non thermal effect of millimeter waves. Electromag Biol Med.

[97] D’Agostino S, Della Monica C, Palizzi E, Di Pietrantonio F, Benetti M, Cannatà D (2018). Extremely high frequency electromagnetic fields facilitate electrical signal propagation by increasing transmembrane potassium efflux in an artificial axon model. Sci Rep.

[98] Ramundo-Orlando A, Longo G, Cappelli M, Girasole M, Tarricone L, Beneduci A (2009). The response of giant phospholipid vesicles to millimeter waves radiation. Biochem Biophys Acta.

[99] Di Donato L, Cataldo M, Stano P, Massa R, Ramundo-Orlando A (2012). Permeability changes of cationic liposomes loaded with carbonic anhydrase induced by millimeter waves radiation. Radiat Res.

[100] Cosentino K, Beneduci A, Ramundo-Orlando A, Chidichimo G (2013). The influence of millimeter waves on the physical properties of large and giant unilamellar vesicles. J Biol Phys.

[101] Manikowska E, Luciani JM, Servantie B, Czerski P, Obrenovitch J, Stahl A (1979). Effects of 9.4 GHz microwave exposure on meiosis in mice. Experientia.

[102] Subbotina TI, Tereshkina OV, Khadartsev AA, Yashin AA (2006). Effect of low-intensity extremely high frequency radiation on reproductive function in Wistar rats. Bull Exp Biol Med.

[103] Volkova NA, Pavlovich EV, Gapon AA, Nikolov OT (2014). Effects of millimeter-wave electromagnetic exposure on the morphology and function of human cryopreserved spermatozoa. Bull Exp Biol Med.

[104] Kesari KK, Behari J (2010). Microwave exposure affecting reproductive system in male rats. Appl Biochem Biotechnol.

[105] Kumar S, Kesari KK, Behari J (2011). Influence of microwave exposure on fertility of male rats. Fertil Steril.

[106] Gapeyev AB, Safronova VG, Chemeris NK, Fesenko EE (1997). Inhibition of the production of reactive oxygen species in mouse peritoneal neutrophils by millimeter wave radiation in the near and far field zones of the radiator. Bioelectrochem Bioenerg.

[107] Gapeyev AB, Yakushina VS, Chemeris NK, Fesenko EE (1998). Modification of production of reactive oxygen species in mouse peritoneal neutrophils on exposure to low-intensity modulated millimeter wave radiation. Bioelectrochem Bioenerg.

[108] Safronova VG, Gabdoulkhakova AG, Santalov BF (2002). Immunomodulating action of low intensity millimeter waves on primed neutrophils. Bioelectromagnetics.

[109] Homenko A, Kapilevich B, Kornstein R, Firer MA (2009). Effects of 100 GHz radiation on alkaline phosphatase activity and antigen–antibody interaction. Bioelectromagnetics.

[110] Gapeyev AB, Kulagina TP, Aripovsky AV, Chemeris NK (2011). The role of fatty acids in anti‐inflammatory effects of low‐intensity extremely high‐frequency electromagnetic radiation. Bioelectromagnetics.

[111] Gapeyev AB, Kulagina TP, Aripovsky AV (2013). Exposure of tumor-bearing mice to extremely high-frequency electromagnetic radiation modifies the composition of fatty acids in thymocytes and tumor tissue. Int J Radiat Biol.

[112] Gapeyev AB, Aripovsky AV, Kulagina TP (2015). Modifying effects of low-intensity extremely high-frequency electromagnetic radiation on content and composition of fatty acids in thymus of mice exposed to X-rays. Int J Radiat Biol.

[113] Rotkovská D, Moc J, Kautská J, Bartonícková A, Keprtová J, Hofer M (1993). Evaluation of the biological effects of police radar RAMER 7F. Environ Health Perspect.

[114] Müller J, Hadeler KP, Müller V, Waldmann J, Landstorfer FM, Wisniewski R (2004). Influence of low power cm-/mm-microwaves on cardiovascular function. Int J Environ Health Res.

[115] Webb SJ, Booth AD (1971). Microwave absorption by normal and tumor cells. Science.

[116] Stensaas LJ, Partlow LM, Bush LG, Iversen PL, Hill DW, Hagmann MJ (1981). Effects of millimeter‐wave radiation on monolayer cell cultures. II. Scanning and transmission electron microscopy. Bioelectromagnetics.

[117] Bellossi A, Dubost G, Moulinoux JP, Himdi M, Ruelloux M, Rocher C (2000). Biological effects of millimeter wave irradiation on mice-preliminary results. IEEE Trans Micro Theory Tech.

[118] Olchowik G, Maj JG (2000). Inhibitory action of microwave radiation on gamma-glutamyl transpeptidase activity in liver of rats treated with hydrocortisone. Folia Histochemica Et Cytobiologica.

[119] Khizhnyak EP, Ziskin MC (1996). Temperature oscillations in liquid media caused by continuous (nonmodulated) millimeter wavelength electromagnetic irradiation. Bioelectromagnetics.

[120] Sarapultseva EI, Igolkina JV, Tikhonov VN, Dubrova YE (2014). The in vivo effects of low-intensity radiofrequency fields on the motor activity of protozoa. Int J Radiat Biol.

[121] Robinette CD, Silverman C, Jablon S (1980). Effects upon health of occupational exposure to microwave radiation (radar). Am J Epidemiol.

[122] Groves FD, Page WF, Gridley G, Lisimaque L, Stewart PA, Tarone RE (2002). Cancer in Korean war navy technicians: mortality survey after 40 years. Am J Epidemiol.

[123] Degrave E, Autier P, Grivegnée AR, Zizi M (2005). All-cause mortality among Belgian military radar operators: a 40-year controlled longitudinal study. Eur J Epidemiol.

[124] Degrave E, Meeusen B, Grivegnée AR, Boniol M, Autier P (2009). Causes of death among Belgian professional military radar operators: a 37‐year retrospective cohort study. Int J Cancer.

[125] Dabouis V, Arvers P, Debouzy JC, Sebbah C, Crouzier D, Perrin A (2016). First epidemiological study on occupational radar exposure in the French Navy: a 26-year cohort study. Int J Environ Health Res.

[126] Hayes RB, Brown LM, Pottern LM, Gomez M, Kardaun JW, Hoover RN (1990). Occupation and risk for testicular cancer: a case-control study. Int J Epidemiol.

[127] Davis RL, Mostofi FK (1993). Cluster of testicular cancer in police officers exposed to hand‐held radar. Am J Ind Med.

[128] Hardell LE, Näsman A, Ohlson CG, Fredrikson MA (1998). Case-control study on risk factors for testicular cancer. Int J Oncol.

[129] Baumgardt-Elms C, Ahrens W, Bromen K, Boikat U, Stang A, Jahn I (2002). Testicular cancer and electromagnetic fields (EMF) in the workplace: results of a population-based case–control study in Germany. Cancer Causes Control.

[130] Walschaerts M, Muller A, Auger J, Bujan L, Guérin JF, Lannou DL (2007). Environmental, occupational and familial risks for testicular cancer: a hospital‐based case‐control study. Int J Androl.

[131] Grayson JK (1996). Radiation exposure, socioeconomic status, and brain tumor risk in the US Air Force: a nested case-control study. Am J Epidemiol.

[132] Santana VS, Silva M, Loomis D (1999). Brain neoplasms among naval military men. Int J Occup Environ health.

[133] Holly EA, Aston DA, Ahn DK, Smith AH (1996). Intraocular melanoma linked to occupations and chemical exposures. Epidemiology.

[134] Stang A, Anastassiou G, Ahrens W, Bromen K, Bornfeld N, Jöckel KH (2001). The possible role of radiofrequency radiation in the development of uveal melanoma. Epidemiology.

[135] La Vecchia CA, Negri E, D’avanzo BA, Franceschi S (1990). Occupation and the risk of bladder cancer. Int J Epidemiol.

[136] Finkelstein MM (1998). Cancer incidence among Ontario police officers. Am J Ind Med.

[137] Fabbro-Peray P, Daures JP, Rossi JF (2001). Environmental risk factors for non-Hodgkin’s lymphoma: a population-based case–control study in Languedoc-Roussillon, France. Cancer Causes Control.

[138] Variani AS, Saboori S, Shahsavari S, Yari S, Zaroushani V (2019). Effect of occupational exposure to radar radiation on cancer risk: a systematic review and meta-analysis. Asian Pac J cancer prev.

[139] Weyandt TB, Schrader SM, Turner TW, Simon SD (1996). Semen analysis of military personnel associated with military duty assignments. Reprod Toxicol.

[140] Hjollund NH, Bonde JP, Skotte J (1997). Semen analysis of personnel operating military radar equipment. Reprod Toxicol.

[141] Schrader SM, Langford RE, Turner TW, Breitenstein MJ, Clark JC, Jenkins BL (1998). Reproductive function in relation to duty assignments among military personnel. Reprod Toxicol.

[142] Velez De La Calle JF, Rachou E, le Martelot MT, Ducot B, Multigner L, Thonneau PF (2001). Male infertility risk factors in a French military population. Hum reprod.

[143] Baste V, Riise T, Moen BE (2008). Radiofrequency electromagnetic fields; male infertility and sex ratio of offspring. Eur J Epidemiol.

[144] Møllerløkken OJ, Moen BE (2008). Is fertility reduced among men exposed to radiofrequency fields in the Norwegian Navy?. Bioelectromagnetics.

[145] De Roos AJ, Teschke K, Savitz DA, Poole C, Grufferman S, Pollock BH (2001). Parental occupational exposures to electromagnetic fields and radiation and the incidence of neuroblastoma in offspring. Epidemiology.

[146] Mageroy N, Mollerlokken OJ, Riise T, Koefoed V, Moen BE (2006). A higher risk of congenital anomalies in the offspring of personnel who served aboard a Norwegian missile torpedo boat. Occup Environ Med.

[147] Baste V, Moen BE, Oftedal G, Strand LA, Bjørge L, Mild KH (2012). Pregnancy outcomes after paternal radiofrequency field exposure aboard fast patrol boats. J Occup Environ Med.

[148] Beard JD, Kamel F (2015). Military service, deployments, and exposures in relation to amyotrophic lateral sclerosis etiology and survival. Epidemiol Rev.

[149] Garaj-Vrhovac V, Gajski G, Pažanin S, Šarolić A, Domijan AM, Flajs D (2011). Assessment of cytogenetic damage and oxidative stress in personnel occupationally exposed to the pulsed microwave radiation of marine radar equipment. Int J Hyg Environ Health.

[150] Mortazavi SM, Shahram TA, Dehghan N (2013). Alterations of visual reaction time and short term memory in military radar personnel. Iran J Public Health.

[151] Singh S, Mani KV, Kapoor N (2015). Effect of occupational EMF exposure from radar at two different frequency bands on plasma melatonin and serotonin levels. Int J Radiat Biol.

[152] Ahlbom A, Green A, Kheifets L, Savitz D, Swerdlow A (2004). ICNIRP standing committee on epidemiology: epidemiology of health effects of radiofrequency exposure. Environ Health Perspect.

[153] Savitz DA (1995). Exposure assessment strategies in epidemiological studies of health effects of electric and magnetic fields. Sci Total Environ.

[154] J‐H Kim S, Ioannides SJ, Elwood JM (2015). Trends in incidence of primary brain cancer in New Zealand, 1995 to 2010. Aust NZ J Public Health.

[155] Karipidis K, Elwood M, Benke G, Sanagou M, Tjong L, Croft RJ (2018). Mobile phone use and incidence of brain tumour histological types, grading or anatomical location: a population-based ecological study. BMJ Open.

